# Disparities in outpatient and inpatient utilization by rural-urban areas among older Mongolians based on a modified WHO-SAGE instrument

**DOI:** 10.1186/s12913-021-07156-y

**Published:** 2021-10-30

**Authors:** Vasoontara Sbirakos Yiengprugsawan, Gantuya Dorj, Jocelyn G Dracakis, Bilegt Batkhorol, Undram Lkhagvaa, Dulamsuren Battsengel, Chimedsuren Ochir, Nirmala Naidoo, Paul Kowal, Robert G Cumming

**Affiliations:** 1 Australian Research Council Centre of Excellence in Population Ageing Research (CEPAR), University of New South Wales, Sydney, Australia; 2 School of Public Health, Mongolian National University of Medical Sciences, Ulaanbaatar, Mongolia; 3 Faculty of Medicine and Health, The University of Sydney, Camperdown, Australia; 4 World Health Organization, Geneva, Switzerland; 5 International Health Transitions, Canberra, Australia

**Keywords:** Aging, Equity, Low and middle-income country, Primary health care, Universal health coverage

## Abstract

**Background:**

Mongolia has made significant progress towards achieving Universal Health Coverage (UHC), but there are still challenges ahead with population ageing and non-communicable diseases (NCDs). The purpose of this study was to investigate patterns and determinants of outpatient and inpatient health service use amongst older people in Mongolia.

**Methods:**

Data were collected using a questionnaire developed for the World Health Organization’s Study on global AGEing and adult health (WHO SAGE). There were 478 participants from rural areas and 497 participants from Ulaanbaatar (further divided into 255 *ger/yurt* district and 242 *apartment* district residents). Multivariable logistic regression analyses were used to investigate determinants of outpatient and inpatient health service use with reported adjusted Odds Ratios (AORs) and 95 % Confidence Intervals (CIs).

**Results:**

Participants were aged 60 to 93 years. About 55 % of respondents used outpatient services in the past 12 months and 51 % used inpatient services in the past three years. Hypertension was the most common reason for health service use. Rural residents had longer travel times and were more likely to incur out-of-pocket expenditure (OOP). Multivariable logistic regression revealed that women were more likely to use outpatient services (AOR 1.88; 1.34-2.63). Compared to *apartment* residents in urban areas, *ger* residents in urban areas were less likely to use outpatient services (AOR 0.54; 0.36-0.83). There was no statistically significant differences in inpatient service by location. Increasing numbers of chronic conditions (1 and 2+ compared to none) were associated with both outpatient (AORs 2.59 and 2.78) and inpatient (AORs 1.97 and 3.01) service use.

**Conclusions:**

This study highlights the needs to address disparities in outpatient service use for rural and urban *ger* populations. Compared with other WHO-SAGE countries, older Mongolians have relatively higher use of inpatient health care services. With a high prevalence of hypertension and an ageing population, efforts to achieve UHC would benefit from reorienting care services towards prevention and primary care management of NCDs to reduce the costs from hospital-based care.

**Supplementary Information:**

The online version contains supplementary material available at 10.1186/s12913-021-07156-y.

## Background

Demographic and epidemiological transitions have resulted in population ageing that poses both opportunities and challenges for low and middle-income countries [1, 2]. Like other countries in the Asia-Pacific region, Mongolia’s population is ageing rapidly [3, 4]. The percentage of the population aged 60 years and over was 7.3 % in 2018 and is predicted to increase to 12.5 % in 2035 and 17.7 % in 2050 [4]. Mean life expectancy at birth has increased substantially over recent years yet the country still experiences the largest discrepancy between males (66 years) and females (74 years) in the East Asia and Pacific region [4, 5]. Chronic non-communicable diseases pose the most predominant health challenges in Mongolia, with very high rates of primary liver cancers and cirrhosis, alongside ischemic heart disease and haemorrhagic stroke due to the high prevalence of hypertension [6–8].

Geo-politically, Mongolia is a lower middle-income country of 3.2 million people and is the second largest landlocked country in the world after Kazakhstan as well as one of the most sparsely populated [9]. Situated between China and Russia, it has been influenced by both powers but has maintained its own strong culture. Approximately half of the total Mongolian population lives in the country’s only large city, Ulaanbaatar, due to internal migration. This rapid urbanising process has occurred over recent decades and as a result, economic and health inequalities exist within urban areas especially between those living in *ger/yurts* and apartment areas [10–12].

Mongolia has made significant progress towards Universal Health Coverage for over 90 % of its population, funded by a mixed general taxation and social insurance scheme [12–14]. A 2016 assessment by the World Health Organization ranked Mongolia’s UHC Index at 63 (out of 100); however, out-of-pocket expenditure contributes 32 % of total health expenditure [15]. Delivery of health services is challenged by very large sparsely populated areas despite increasing urbanisation. Primary health care is delivered by Family Health Centres (FHC) in city districts and by Soum Health Centres (SHC) in rural areas. Most tertiary and all specialised hospitals are located in the capital city of Ulaanbaatar and, if referred from primary care providers, public secondary and tertiary services can be accessed free of charge. However, many patients prefer to present directly to secondary and tertiary services without appropriate referrals resulting in out-of-pocket expenditure and inequity in health service utilization [11–13]. Co-payments have also been cited as an informal feature imposed by many providers across both primary and higher levels of care in rural areas [11–13]. In addition to health system related factors, education and income inequalities have been reported to contribute to disparities in health status and health service utilization [15, 16].

The purpose of this study was to investigate patterns and determinants of outpatient and inpatient health service use amongst older people in Mongolia. Our analyses took into account urban-rural differences and findings could inform public health practitioners and policy makers on Universal Health Coverage and equity of service use for older Mongolians.

## Methods

### Data collection

Data were collected using questionnaires developed for the World Health Organization Study on global AGEing and adult health (WHO SAGE) [17, 18]. In the context of this study, a few response categories (ethnicity, religion, and place of residence) were modified to correspond to Mongolia. No other changes to wording of questions were made. The survey team participated in a one-week training workshop led by an experienced WHO SAGE researcher. The survey was implemented between November 2017 and April 2018. Data were collected through face-to-face interviews using structured questionnaires and clinical assessments and took between 45 min and 90 min to complete.

### Selection of study subjects

The survey sample included respondents selected from Ulaanbaatar, the only large city in Mongolia, and two rural provinces (*aimag*), Dundgovi and Uvurkhangai, purposively selected from the 21 *aimag* in Mongolia because of their proximity to Ulaanbaatar. The sampling procedures differed between Ulaanbaatar and the rural *aimag*. Ulaanbaatar is comprised of nine districts (*düüreg*) subdivided into *khoroo*. Four (Bayanzurkh, Chingeltei, Khan-Uul and Sukhbaatar) of the six central districts and one (Nailakh) of the three peripheral districts were randomly selected. Two *khoroo* were randomly selected from each of the five study districts, one *ger/yurts* area and one *apartment* area. New rural migrants to Ulaanbaatar often live-in settlements of traditional *ger* which are distinct from areas of more established dwellings and other infrastructure. Fifty people aged 60 years and over were randomly selected from each of the 10 study *khoroo*. Mongolia’s rural *aimag* are divided administratively into *soum*. Four study *soum* were purposively selected by population size, the two with the largest (Saintsagaan and Arvaikheer) and second largest (Erdenelei and Khrakhorin) population from each of the two study *aimag*. In each of the four study *soum*, 125 people aged 60 years and older were randomly selected for the study.

In total, the final sample size was 975 people aged 60 years and older, with approximately half from urban and half from rural areas. The participation rate was 98.3 % (99.4 % in Ulaanbaatar and 96.8 % in the rural areas). With close to 500 participants in each group, the study has 80 % power (p value 0.05) to detect a 10 % prevalence difference in health service use which is of public health policy importance and also evident in other WHO SAGE countries [19] as well as between rural and urban areas in Mongolia [11, 14].

### Variables of interest

The SAGE Mongolia individual questionnaire included modules on sociodemographic characteristics, health status, health service utilization and health related expenditures.

- Demographic and socioeconomic variables included sex, age, marital status, ethnicity, region, educational attainment, economic tertile (derived from annual income), and residence in rural, urban *ger* and urban *apartment* areas.
- For outpatient service use, questions included: “Over the last 12 months, did you receive any health care NOT including overnight stay in hospital or long-term care facility?” “Where did you go most often when you felt sick or needed to consult someone about your health” [public clinic including FHC and SHC, public hospital, private clinic, private hospital]?
- For inpatient health service use, questions included: “In the last 3 years, have you ever stayed overnight in a hospital or other healthcare e.g. long-term care facility?” with corresponding categories [public hospital, private hospital, and other]. The most common reasons for either outpatient or inpatient service use were broadly categorised into communicable/maternal/acute conditions, chronic joint / arthritis, heart problems, hypertension, liver cirrhosis, liver cancer and other cancers, diabetes, other internal medicine conditions, and others. The number of chronic conditions (none, 1, 2+) including diabetes, hypertension, asthma, lung diseases, heart conditions, arthritis were included as proxy for health care needs.
- Questions relating to outpatient and inpatient health service travel and payment included: mode of travel (private vehicle, taxi, walk, public transport), duration of commute and sources of payment (self, spouse, son/daughter, other family, insurance, free of charge). All respondents were asked about their satisfaction levels with how health services are conducted overall and ratings of how well services involve patients in decision making. Responses were grouped into satisfied/very satisfied, neither, dissatisfied/very dissatisfied.

### Statistical analysis

Data were entered into SPSS version 17 [20] and analyses conducted using Stata version 19 [21]. Statistical differences were assessed by chi-square (χ^2^) to show a relationship between categorical variables by sex and urban-rural areas and p-value of 0.05 was used to assess its statistical significance level. Selected variables for analyses were based on previous literature on factors relating to health care utilization in WHO SAGE data [19, 22]. In addition, correlation tests were conducted for explanatory variables and the largest being 0.37 between education and rural-urban areas, there is no evidence of multicollinearity in the variables included in the model. Multivariable logistic regression analyses were used to investigate determinants of outpatient and inpatient health services use, reporting adjusted Odds Ratios (AORs) and 95 % Confidence Intervals (CIs).

## Results

Participants ranged in age from 60 to 93 years. There were 478 participants from rural areas and 497 participants from Ulaanbaatar (comprised of 255 *ger* and 242 *apartment* residents). Compared with the rural population, the urban population had a greater predominance of females and widows, higher levels of educational attainment, and far greater representation in the uppermost economic tertile [Table 1]*.* Within urban areas in Ulaanbaatar, a higher proportion of *apartment* residents were aged 80 years and older and were of higher socioeconomic status (education and economic tertile) as compared with *ger* residents. Notably, greater ethnic and religious diversity of the urban *ger* population reflects the presence of groups who migrated from far western Mongolia.

**Table 1:** Demographic and socioeconomic characteristics in Mongolia, 2017-2018

Attributes		Percent (***n***=975)		
		Rural areas (***n***=478)	Urban areas	
			***ger (n=255)***	***apartment (n=242)***
**Sex**	Male	43.9 (210)	34.9 (89)	33.5 (81)
	Female	56.1 (268)	65.1 (166)	66.5 (161)
**Age Group**	60-69	62.6 (299)	69.4 (177)	47.1 (114)
	70-79	28.7 (137)	27.8 (71)	35.1 (85)
	80+	8.8 (42)	2.8 (7)	17.8 (43)
**Marital Status**	Married/Cohabiting	63.8 (305)	51.8 (132)	53.7 (130)
	Not married*	5.4 (26)	3.1 (8)	2.9 (7)
	Widowed	30.8 (147)	45.1 (115)	43.4 (105)
**Ethnicity**	Mongol^†^	99.4 (475)	86.7 (221)	94.2 (228)
	Kazak‡	0.2 (1)	11.8 (30)	3.7 (9)
	Other	0.4 (2)	1.6 (4)	2.1 (5)
**Religion**	None	17.2 (82)	35.7 (91)	35.5 (86)
	Buddhist	81.2 (388)	57.7 (147)	60.7 (147)
	Other	1.0 (5)	6.7 (17)	3.3 (8)
	Missing	(3)	(0)	(1)
**Educational Attainment**	No formal education	5.1 (24)	1.6 (4)	1.6 (4)
	Primary school	33.8 (159)	7.2 (18)	5.0 (12)
	Secondary school	22.8 (107)	29.5 (74)	19.0 (46)
	High school	12.6 (59)	15.5 (39)	19.4 (47)
	Tertiary or higher	25.7 (121)	46.2 (116)	55.0 (133)
	Missing	(8)	(4)	(0)
**Economic Tertiles**	Tertile 1 (lowest)	41.6 (162)	29.4 (68)	24.9 (48)
	Tertile 2	39.6 (154)	29.0 (67)	22.3 (43)
	Tertile 3 (highest)	18.8 (73)	41.6 (96)	52.9 (102)
	Missing	(89)	(24)	(49)

*includes never married (*n*=12) and separated/divorced (*n*=14)

^†^includes Khalkh, Dorwod, Bayad, Buriad, Zachin, Dariganga

‡ includes Kazak and the minority Turkic ethnicity Urianhai

Approximately half of all respondents used outpatient health care in the past year [Table 2]. Use of outpatient services was lower among people living in urban *ger* areas (47.8 %) and rural areas (53.4 %) than among people in urban *apartment* areas (66.5 %). There was no statistically significant difference in outpatient service use between rural and urban *ger* areas but these were both statistically significantly lower than urban *apartment* areas. The use of public clinic facilities (FHC and SHC) was most commonly reported, followed by public hospital outpatient clinics across all groups. For inpatient healthcare utilization, approximately reported needing overnight care in the past three years [Table 2]*.* Overall, there was no statistically significant difference in the public versus private choices of inpatient health service use between urban and rural areas.

**Table 2:** Health service utilization by sex and urban-rural areas in Mongolia, 2017-2018

% (n)	Overall	Sex		Residence		
		***Male***	***Female***	***Rural***	***Urban - ger***	***Urban - apartment***
**Outpatient visits in the past year**						
Yes	55.2 (538)	45.3 (172)	61.5 (366)	53.4 (255)	47.8 (122)	66.5 (161)
No	44.8 (437)	54.7 (208)	38.5 (229)	46.7 (223)	52.2 (133)	33.5 (81)
*Type of outpatient facility*						
Public clinic, including health centers	62.8 (337)	59.9 (103)	64.1 (234)	55.5 (141)	68.8 (84)	69.6 (112)
Public hospital	23.8 (128)	27.3 (47)	22.2 (81)	28.7 (73)	19.7 (24)	19.3 (31)
Private facilities	11.2 (60)	10.5 (18)	11.5 (42)	11.0 (28)	11.5 (14)	11.3 (18)
Other	2.3 (12)	2.3 (4)	2.2 (8)	4.7 (12)	n/a	n/a
Inpatient stays in the past 3 years*						
Yes	51.1 (498)	46.0 (175)	54.3 (323)	49.2 (235)	52.2 (132)	53.7 (130)
No	48.9 (477)	53.9 (205)	45.7 (272)	50.8 (243)	47.8 (122)	46.3 (112)
*Type of inpatient facility*						
Public hospital	83.3 (330)	90.3 (121)	80.1 (193)	84.2 (171)	83.0 (83)	81.7 (76)
Private hospital	13.6 (54)	6.7 (9)	17.8 (43)	11.3 (23)	15.0 (15)	17.2 (16)
Other	3.0 (12)	3.0 (4)	2.1 (5)	4.4 (9)	2.0 (2)	1.1 (1)
Reasons for health service use**						
Acute or communicable conditions	4.8	4.0	5.2	4.6	4.5	5.3
Chronic joint pain ‎/ arthritis	14.9	10.9	16.9	16.2	11.1	15.9
Heart problems	12.8	15.2	11.5	11.8	15.2	12.4
Hypertension	31.6	32.6	31.1	32.1	30.3	31.9
Liver cirrhosis, liver cancer, other cancers	3.2	4.3	2.6	2.6	4.0	3.5
Diabetes	2.0	2.2	1.9	0.8	1.5	4.4
Other internal medicine†	5.8	3.6	6.5	5.4	6.1	6.2
Other‡	25.0	27.2	24.2	26.5	27.3	20.4
**Chronic conditions**						
0	18.7 (184)	27.1 (103)	12.3 (73)	24.9 (119)	13.3 (34)	9.5 (23)
1	31.0 (305)	32.1 (122)	30.8 (183)	32.9 (157)	31.8 (81)	27.7 (67)
2+	50.2 (494)	40.8 (155)	56.9 (339)	42.3 (202)	54.9 (140)	62.8 (152)

* Out of 498 respondents who had inpatient stays, 8 were reported in long-term care facility. ** either inpatient or outpatient; †includes stomach, pancreas, kidney, gynaecological, prostate, bladder, haematological and nutritional disorders; generalised visceral or muscular pain; ‡ includes surgery, injuries, regular check-ups, breathing problems, sleep disorders, fatigue, allergies, hearing, vision, dental, neuropathy

The most common condition leading to either outpatient or inpatient service use was hypertension [Table 2]. Notably, crude proportions of chronic joint and muscular pain were more common among females and hypertension among males. Reasons for health service use differed by sex (χ^2^*p*=0.016) as well as between rural and the two urban areas (χ^2^ < 0.001). Approximately 30 % of correspondents reported one chronic condition, 30 % reported at least two chronic conditions, 15 % with three conditions, and 4 % with four conditions and over.

Regarding health service accessibility, the most common mode of travel to outpatient services was public transport, particularly among urban (69.5 %) compared to rural residents (42.1 %), while using private vehicles was the most common mode of travel to in-patient services in both rural and urban areas [Table 3]. Travel times over an hour for outpatient and inpatient health service use was significantly higher in rural areas. Urban residents accessed a statistically significant higher proportion of free outpatient and inpatient services than rural residents (both χ^2^*p*<0.001) [Table 3].

**Table 3:** Accessibility and payment for health services and general satisfaction with health system services [percent, (n)] by sex and urban-rural areas in Mongolia, 2017-2018

	Sex		Residence		
	***Male***	***Female***	***Rural***	***Urban - ger***	***Urban - apartment***
**Outpatient service use**					
*Travel mode*					
Private vehicle	34.2 (53)	22.6 (75)	37.7 (86)	15.3 (17)	16.9 (25)
Public Transport	48.4 (75)	60.5 (201)	42.1 (96)	63.9 (71)	73.7 (109)
Taxi	7.7 (12)	4.5 (15)	5.3 (12)	11.7 (13)	6.1 (9)
Walk	9.7 (15)	12.4 (41)	14.9 (34)	9.0 (10)	3.4 (5)
*Travel time*					
<1 hour	86.9 (140)	87.9 (312)	82.1 (197)	94.2 (113)	91.0 (142)
≥1 hour	13.0 (21)	21.1 (43)	17.9 (43)	5.8 (7)	9.0 (14)
*Payment*					
Self or spouse	11.4 (18)	7.4 (26)	11.9 (27)	6.6 (8)	5.6 (9)
Son/daughter or other family	4.4 (7)	2.9 (10)	3.1 (7)	3.3 (4)	3.7 (6)
Insurance	6.3 (10)	4.3 (15)	10.2 (23)	0.8 (1)	0.6 (1)
Free	77.9 (123)	85.4 (299)	74.8 (169)	89.3 (108)	90.1 (145)
**Inpatient service use**					
*Travel mode*					
Private vehicle	51.8 (72)	45.9 (117)	54.2 (109)	40.0 (40)	43.0 (40)
Public Transport	9.4 (13)	14.1 (36)	6.5 (13)	22.0 (22)	15.1 (14)
Taxi	14.4 (20)	20.8 (53)	13.4 (27)	25.0 (25)	22.6 (21)
Walk	21.6 (30)	15.3 (39)	22.9 (46)	2.0 (2)	6.5 (6)
Ambulance	2.9 (2)	3.9 (10)	3.0 (6)	11.0 (11)	12.9 (12)
*Travel time*					
<1 hour	79.4 (108)	73.5 (186)	71.5 (143)	78.4 (76)	81.5 (75)
≥1 hour	20.6 (28)	26.5 (67)	28.5 (57)	21.7 (21)	18.5 (17)
*Payment*					
Self or spouse	20.0 (28)	17.5 (45)	23.0 (47)	12.0 (12)	15.0 (14)
Son/daughter or other family	2.9 (4)	8.2 (21)	4.9 (10)	7.0 (7)	8.6 (8)
Insurance	7.1 (10)	6.6 (17)	9.3 (19)	6.0 (6)	2.2 (2)
Free	70.0 (98)	67.7 (174)	62.7 (128)	75.0 (75)	74.2 (69)
**General satisfaction with health system services**					
*Satisfied/very satisfied*	71.3 (271)	67.4 (401)	75.9 (363)	61.2 (156)	63.2 (153)
*Neither*	21.3 (81)	24.0 (143)	21.1 (101)	25.9 (66)	23.6 (57)
*Dissatisfied/very dissatisfied*	7.4 (28)	8.6 (51)	2.9 (14)	12.9 (9)	13.2 (32)
**Service involvement of patient in decision-making**					
Satisfied/very satisfied	46.8 (178)	46.1 (274)	50.2 (240)	43.5 (111)	41.7 (101)
Neither	39.2 (149)	38.5 (229)	43.1 (207)	34.5 (88)	34.3 (83)
Dissatisfied/very dissatisfied	13.9 (53)	15.5 (92)	6.5 (31)	21.9 (56)	23.9 (58)

When asked about satisfaction with the systems of health service delivery in Mongolia and how well services involve patients in decision-making, males and females reported similar responses [Table 3]. However, statistically significantly higher proportions of urban compared with rural residents were dissatisfied/very dissatisfied. There was no difference in reported satisfaction between urban *ger* and *apartment* areas.

In the adjusted multivariable logistic regression analyses [Table 4], females were almost double more likely than males to use outpatient health services. Older adults in the lowest economic tertile were statistically less likely to use outpatient services (AORs 0.63). Increasing numbers of chronic conditions (1 and 2+ compared to none) were associated with both outpatient (AORs 2.59 and 2.78) and inpatient (AORs 1.97 and 3.01) service use. There was no difference in use of outpatient and inpatient health services between rural residents and the combined urban population of *ger* and *apartment* areas. However, when further compared to *apartment* residents in urban areas, *ger* residents in urban areas were much less likely to use outpatient services (AORs 0.54).

**Table 4:** Fully adjusted multivariable logistic regression on determinants of outpatient and inpatient service use in Mongolia, 2017-2018

Attributes		Adjusted Odds Ratios [95% Confidence Interval]	
		Outpatient use	Inpatient use
**Sex**			
	Male	Reference	Reference
	Female	**1.88** [1.34; 2.63]	1.27 [0.91-1.77]
**Age group**			
	60-69	Reference	Reference
	70-79	1.00 [0.56; 1.80]	0.86 [0.48; 1.52]
	80+	1.65 [0.55; 4.94]	0.73 [0.25; 2.15]
**Marital status**			
	Married/Cohabiting	Reference	Reference
	Not married/Divorced	1.44 [0.68; 3.05]	**0.45** [0.21; 0.96]
	Widowed	0.92 [0.64; 1.33]	0.91 [0.63; 1.30]
**Education**			
	Primary school or below	Reference	Reference
	Secondary school	1.02 [0.66; 1.60]	1.18 [0.75; 1.82]
	High school	0.96 [0.65; 1.41]	1.32 [0.99; 1.94]
	Tertiary or higher	0.85 [0.54; 1.33]	0.88 [0.81; 1.69]
**Economic tertile**			
	Tertile 1 (lowest)	**0.63 [0.41; 0.94]**	1.32 [0.89; 1.98]
	Tertile 2	0.73 [0.50; 1.06]	1.17 [0.81; 1.70]
	Tertile 3 (highest)	Reference	Reference
**Urban-rural areas**			
	Rural areas	0.81 [0.53; 1.23]	1.12 [0.74; 1.67]
	Urban areas		
	*ger*	**0.54 [0.36; 0.83]**	1.08 [0.72; 1.63]
	*apartment*	Reference	Reference
**Chronic conditions**			
	0	Reference	Reference
	1	**2.59 [1.66; 4.04]**	**1.97 [1.28; 3.07]**
	2+	**2.78 [1.82; 4.25]**	**3.01 [1.98; 4.57]**

Further stratified analyses by urban and rural areas reveal that females in rural areas were twice more likely than males to use outpatient services (Supplement Table 1). Chronic conditions were strong factors associated with both outpatient and inpatient service use in urban as well as rural areas. Notably, additional multivariable logistic regression analyses amongst outpatient and inpatient service users confirm that rural residents were over three times more likely to pay for outpatient services compared to urban-*apartment* residents (Supplement Table 2).

## Discussion

This study investigates patterns and determinants of outpatient and inpatient health service use among older people in Mongolia. Slightly more than half of respondents reported outpatient health service use in the past year. Urban *ger* area residents and rural residents were significantly less likely to have used outpatient services than urban *apartment* area residents.

The main disadvantages for rural residents were longer travel times than respondents living in urban areas for both outpatient and inpatient services and a higher proportion reporting out-of-pocket expenditure by self or spouse to pay for health services in rural areas. Notably, urban residents were three times more likely to express dissatisfaction with how health services are conducted and their involvement in their health care decision-making.

Amongst older persons in low and middle-income countries in WHO SAGE, Mongolia had lower use of outpatient service (55 %) compared to 60–70 % in China, Ghana, Russia, South Africa and 80 % in India [17]. However, Mongolia had a much higher proportion of older people using inpatient services in the past three years (over 50 %) among respondents aged 60 years and over. The corresponding proportions in the immediate neighbouring countries for respondents aged 50 years and over were 22 % in China and 30 % in the Russian Federation [19, 24]. Other countries in WHO SAGE including Ghana, India, Mexico, South Africa all reported less than 20 % using inpatient services in the past three years. Notably, both Mongolia and Russia have continued high hospital use as part of the legacy of the Union of Soviet Socialist Republics health system between 1922 and 1991 [25]. These high health service utilization rates and a high number of hospital beds have been reported in former Soviet Union countries [26].

These results are similar to other countries where WHO SAGE surveys have been conducted, notably, respondents with two or more chronic conditions were twice or three times more likely to use health care services [20]. As well, aligned with other studies, older women were more likely than men to use outpatient services [19]. A plausible explanation is that men are more likely to delay accessing care, especially for less urgent chronic health conditions. Another explanation for higher use of outpatient use by women could be that older women have a higher prevalence of chronic disease. In the case of Mongolia, a chronic disease risk factor surveillance study found that females aged 55-64 years had a higher prevalence of NCDs than men (42.6 % vs. 36.1 %) [23].

Within Mongolia, there are significant disparities between urban and rural areas in terms of distribution of health facilities and human resources, which are more concentrated in urban centres [27, 28]. We found that older people living in urban *ger* areas had a much lower use of outpatient services than older people in *apartment* areas. Rural to urban migrants living in the outskirts of urban areas, in particular *ger* residents, face limited services in Family Health Centres (FHC) while the rural poor are strongly dependent on *Soum* Health Centres (SHC) with limited or no access to higher levels of health services [30]. Notably, this study reported significant differences in dissatisfaction with health services and involvement in health care decision making. Hence, policy design and service delivery would benefit from engaging older Mongolians to better understand their preferences and barriers to health service use. Future studies could also provide insights into motivations behind health care seeking behaviours among older Mongolians as the health system evolves.

Unofficial self-referrals to secondary and tertiary care have been well documented and are a fundamental challenge to the Mongolian health system [11–13]. Higher income individuals tend to bypass FHCs in urban areas and self-refer to higher level tertiary and specialised hospitals as they can afford the penalty fee leaving lower income urban-*ger* residence, especially rural to urban migrants with limited options [11–13]. Several factors relating to bypassing local primary health care have been attributed to unavailability of diagnostic capacities, treatment options and essential medicines [11, 13]. In order to strengthen and reorient services towards primary health, greater funding would enhance the range of service facilities and attract skilled health professionals in remote areas [12, 13].

Our data support previous findings from the Mongolian Household Socio-Economic Survey data 2007/2008, 2012, and 2018 that free access to health services does not guarantee equitable access [11, 13, 30]. Reasons given for not seeking treatment in the 2018 survey include the facility was too far or could not afford travel highlighting the influential role of geographic challenges compounded by a weak transport infrastructure [30]. Limited and scattered infrastructure in rural areas further contribute to widening geographical health service inequalities [11, 13, 29, 30].

Additionally, the Mongolian State Policy on Health (2017-2026) has promoted the use of mobile health screening or M-Health in combination with home visits and primary health care services in 21 provinces across Mongolia, including in Ulaanbaatar [27, 31]. These M-Health services help to not only reach out to disadvantaged communities, including migrants in urban-*ger* areas but also address geographical barriers for low-income rural residents and nomadic populations in remote areas. Examples of M-health screening services include health promotion for NCDs (e.g. monitoring cholesterol and blood sugar), preventative ultrasound and electrocardiogram examinations, and outreach services during COVID-19 [27, 31].

The main strength of the study lies in its international design using the WHO SAGE study materials and methods for research on the health of older people in low and middle-income countries [17, 18]. Our study also had a very high participation rate similar to other SAGE countries in Asia (85 % in India and 93 % in China) [18]. This study is the most comprehensive population-based study of the health of older people in Mongolia and complements previous NCD risk factor studies that used an upper age cut-off at 65 years [23]. There are study limitations that should be considered in interpreting results. The two rural *aimag* were purposively selected for logistic reasons and are relatively close to Ulaanbaatar. Greater rural-urban disparities may have been identified if more remote *aimag* had been studied. The study analysis has taken into account some individual-level determinants of health but not area-level determinants, including the influence of lifestyle and living environment, and cultural resources such as social and familial support on health and health service use [10].

Primary health care is a cornerstone of UHC and sustainable development in many countries [32]. Evidence from Thailand, Sri Lanka and Malaysia has shown that availability and proximity of access to primary health care could favour older persons [33]. Recent evidence in Mongolia has reported the transformation of FHC and SHC under way towards health promotion (219 FHC and 273 SHC, 41.8 % and 38.6 % for outpatient visits for preventive purposes). According to the Asian Development Bank’s three decades of partnership in Mongolia, primary health services might not yet meet expectations on the range and quality of services and will continue to require both financial and human resources as well as effective referral mechanisms across health services [34, 35].

## Conclusions

With Mongolia’s population ageing, the demand for health services will increase and may put further pressures on health equity for older people living in rural and urban *ger* areas. Findings also reveal that older Mongolians have relatively higher use of inpatient health care services and hence efforts to achieve UHC would benefit from reorienting care services towards prevention and primary care management of NCDs to facilitate accessibility and reduce the costs incurred from using hospital-based care.

## Supplementary information

**Additional file 1.**

**Additional file 2.**

## Data Availability

The datasets in the current study are not publicly available. Data, however, are available from the authors upon reasonable request.

## Abbreviations

AORs: Adjusted Odds Ratios
CIs: Confidence Intervals
FHC: Family Health Centres
NCDs: non-communicable diseases
OOP: Out-of-pocket expenditure
SHC: *Soum* Health Centres
UHC: Universal Health Coverage
WHO SAGE: World Health Organization Study on global AGEing and adult health
