# Supplementary material for: Disparities in outpatient and inpatient utilization by rural-urban areas among older Mongolians based on a modified WHO-SAGE instrument
Source: BMC Health Serv Res. 2021 Oct 30;21:1183. doi: 10.1186/s12913-021-07156-y (PMC8556801; doi:10.1186/s12913-021-07156-y)
Supplement: Supplementary file 1 — Additional file 1. [file 12913_2021_7156_MOESM1_ESM.docx]

STUDY ON GLOBAL AGEING AND ADULT HEALTH

Mongolia – 2017

Individual Questionnaire

Table of Contents

[Contact Record – Individual or Proxy Respondent 2](#_Toc494302598)

[Section 1000: Socio-Demographic Characteristics 4](#_Toc494302599)

[Section 2000: Health State Descriptions 8](#_Toc494302600)

[Section 2500: Anthropometrics, Performance Tests and Biomarkers 12](#_Toc494302601)

[Section 3000: Risk Factors and Preventive Health Behaviours 21](#_Toc494302602)

[Section 4000: Chronic Conditions and Health Services Coverage 26](#_Toc494302603)

[Section 5000: Health Care Utilization 35](#_Toc494302604)

[Section 6000: Social Networks 46](#_Toc494302605)

[Section 7000: Subjective Well-Being and Quality of Life 48](#_Toc494302606)

[Section 9000: Interviewer Assessment 59](#_Toc494302607)

Contact Record – Individual or Proxy Respondent

| Q1000a. Interviewer I.D.  Q1000b. Contact with:  1=Individual respondent  2=Proxy respondent  5=No one  Q1000c. Result code  01=Completed Interview (interview is accepted and conducted – this includes interview and body measurement, performance tests and blood sample)  02=Partial Interview (interview is partially completed and person will not be contacted anymore).  03=Respondent contacted-initial refusal  04=Respondent contacted-uncertain about interview  05=Resistance/refusal by respondent  06=Final refusal by respondent  07=Final refusal by other household member  08=Unable to locate respondent  09=No interview because respondent is not eligible: less than 18, mentally unfit or too ill.  10=Language barrier  11=House is vacant or household occupants are elsewhere (seasonal vacancy, other residence)  12=Unsafe or dangerous area or no access to respondent  13=Deceased respondent  14=Respondent in institution: jail, hospital and not accessible | 1  2  5  01  02  03  04  05  06  07  08  09  10  11  12  13  14 |
| --- | --- |

| Q1001 | *Interviewer:*  *Does the respondent have obvious cognitive limitations that prevent him/her from being interviewed?* | 1. Yes ………………….🡺 2. No | Q1005 |
| --- | --- | --- | --- |
| I would like to start by asking you some background questions before asking you questions about your health. This information is confidential and you will not be identified individually or without your consent.  One of the issues we are exploring in this study is the memory problems that some older persons can have. I know these questions may be sensitive or difficult to answer, but please try to provide an answer. I would like to start by asking you two questions about your memory. | | | |
| Q1002 | How would you best describe your memory at present? Is it very good, good, moderate, bad or very bad? | 1. Very good 2. Good 3. Moderate 4. Bad 5. Very bad |  |
| Q1003 | Compared to 12 months ago, would you say your memory is now better, the same or worse than it was then? | 1. Better 2. Same 3. Worse |  |
| Q1004 | Interviewer: | 1. No reason to think Respondent has any cognitive limitations..🡺 2. Cognitive limitations or health problems, proxy………………🡺 | Individual  Consent form and Individual Q  Q1005 |
| Q1005 | *Interviewer:*  *We would like to ask someone who knows the respondent a few questions about the respondent's memory and health.*  *Who is the proxy?* | 1. Spouse ……………………....🡺 2. Non-spouse……………….….🡺 | Proxy Q  Proxy Q |
|  | *INTERVIEWER: GO TO PROXY CONSENT FORM*  *ADMINISTER Q02501-Q2503 Q2525-Q2546 Q2549-Q2554 TO RESPONDENT*  *THEN ADMINISTER PROXY QUESTIONNAIRE TO PROXY RESPONDENT* | | |

Section 1000: Socio-Demographic Characteristics

Time Begin :

| Q1006 | *Household ID* | | |  | |  |
| --- | --- | --- | --- | --- | --- | --- |
| Q1007 | *Person (HH member) number from HH roster (number from column)* | | |  | |  |
| Q1008 | What is your mother tongue?  By mother tongue, we mean the language you learned first, the language that you can express yourself fully in, or voluntarily identify with. | | | 1. Mongolian 2. Russian 3. English 4. Chinese   *7 Other, specify:* | |  |
| Q1009 | *Interviewer:*  *Record sex of the respondent* | | | 1. *Male* 2. *Female* | |  |
| Q1010 | What day, month and year were you born?  *DD / MM / YYYY*  *Check birth certificate if available.* | | | //  *-8 DK* | |  |
| Q1011 | How old are you now*?*  *INTERVIEWER: This would be age at last birthday. If don't know - probe.* | | | *Age in years* | |  |
| Q1012 | What is your current marital status? | | | 1. *Never married* *……………………..🡺* 2. *currently married ………………..🡺* 3. *cohabiting ………………………….****🡺*** 4. *separated/divorced ……………….* ***🡺*** 5. *widowed …………………………….* ***🡺*** | | *Q1015*  *Q1014*  *Q1014*  *Q1013*  *Q1013* |
| Q1013 | For how many years have you been separated, divorced or widowed?  *INTERVIEWER: if less than 1 year, enter "00"* | | | *Number of years …………….****🡺***  *-8 DK ………………………🡺* | | *Q1015*  *Q1015* |
| Q1014 | For how many years have you been married or living together?  *INTERVIEWER: if less than 1 year, enter "00"* | | | *Number of years*  *-8 DK* | |  |
| Q1015 | Have you ever been to school? | | | 1. *Yes* 2. *No ……………………………….🡺* | | *Q1018* |
| Q1016 | | What is the highest level of education that you have completed? | 1. Less than primary school 2. Primary school completed 3. Secondary school completed 4. High school (or equivalent) completed 5. College/Pre-university/University completed 6. Post graduate degree completed | |  | |
| Q1017 | | How many years of school, including higher education have you completed? | *Number of years*  *-8 DK* | |  | |
| Q1018 | | What is your background or ethnic group? | 1. Khalkh 2. Kazak 3. Dorvod 4. Bayad 5. Buryat-Bouriates 6. Zakhchin 7. Dariganga 8. Uriankhai   87 Other, specify*:* | |  | |
| Q1019 | | Do you belong to a religious denomination?  *INTERVIEWER: allow the respondent to reply without reading categories. Clarify as needed.* | 1. *No, none* 2. *Buddhism* 3. *Chinese traditional religion* 4. *Christianity (including Roman Catholic, Protestant, Orthodox, other)* 5. *Hinduism* 6. *Islam* 7. *Other , specify:*   *9 Refused* | | | |
| Q1020 | | Have you always lived in this village/town/city? | 1. Yes …………………………..🡺 2. No | | *Q1024a* | |
| Q1021 | | How long have you been living (continuously) in this area?  *Interviewer: If less than 1 year, enter “00”.* | *Years*  *-8 DK* | |  | |

| Q1022 | Where were you living before? | 1. In same community/locality/neighborhood 2. In another city in this region 3. In another rural area in this region 4. In another city outside this region but in Mongolia 5. In another rural area outside this region but in Mongolia 6. Outside Mongolia in a city   7 Outside Mongolia in a rural area |
| --- | --- | --- |
| Q1023 | Where have you lived for most of your adult life (18+ years)? | 1. In same community/locality/neighborhood 2. In another city in this region 3. In another rural area in this region 4. In another city outside this region but in Mongolia 5. In another rural area outside this region but in Mongolia 6. Outside Mongolia in a city 7. Outside Mongolia in a rural area |
| Q1024 | Where did you live for most of your childhood (before age 10 years)? | 1. In same community/locality/neighborhood 2. In another city in this region 3. In another rural area in this region 4. In another city outside this region but in Mongolia 5. In another rural area outside this region but in Mongolia 6. Outside Mongolia in a city 7. Outside Mongolia in a rural area |
| Q1024a | Where were you born? | 1. In same community/locality/neighborhood 2. In another city in this region 3. In another rural area in this region 4. In another city outside this region but in Mongolia 5. In another rural area outside this region but in Mongolia 6. Outside Mongolia in a city 7. Outside Mongolia in a rural area |

NEW: Before we move onto the next section, I would like to ask you a few questions about your [*biological*] parents. I would like to know about their level of education and main occupation.

Follow-up: The last time we spoke to you, we asked questions about your mother’s and father’s education and occupations. We have an additional question about their occupation when you were a child.

Let’s start with your mother.

| Q1025 | Was your mother ever employed? | 1. *Yes* 2. *No …………………………..🡺* | *Q1028* |
| --- | --- | --- | --- |
| Q1026 | Who is/was your mother’s main employer over her working life? | 1. Public sector (Government) 2. Private sector (For profit or not for profit) 3. Self-employed 4. Informal employment |  |
| Q1027 | What is/was her main occupation over her working life?  *INTERVIEWER:*  *Use ILO occupation coding and clarify where needed to get to four-digit occupation code* | __________________________________   \|  \|  \|  \|  \| \| --- \| --- \| --- \| --- \| |  |
| *Q1027A* | What was your mother’s main occupation when you were about 10 years old?  *INTERVIEWER:*  *Use ILO occupation coding and clarify where needed to get to four-digit occupation code* | __________________________________   \|  \|  \|  \|  \| \| --- \| --- \| --- \| --- \| |  |

| Q1028 | What is the highest level of education that she completed? | 1. *No formal education* 2. *Less than primary school* 3. *Primary school completed* 4. *Secondary school completed* 5. *High school (or equivalent) completed* 6. *College/Pre-university/University completed* 7. *Post graduate degree completed*   *8 DK* |  |
| --- | --- | --- | --- |

Now if you would please tell me about your father.

| Q1029 | Was your father ever employed? | 1. *Yes* 2. *No …………………………..🡺* | *Q1032* |
| --- | --- | --- | --- |
| Q1030 | Who is/was your father’s main employer over his working life? | 1. Public sector (Government) 2. Private sector (For profit or Not for profit) 3. Self-employed 4. Informal employment |  |
| Q1031 | What is/was his main occupation over his working life?  *INTERVIEWER:*  *Use ILO occupation coding and clarify where needed to get to four-digit occupation code* | _________________________________   \|  \|  \|  \|  \| \| --- \| --- \| --- \| --- \| |  |
| *Q1031A* | What was your father’s main occupation when you were about 10 years old?  *INTERVIEWER:*  *Use ILO occupation coding and clarify where needed to get to four-digit occupation code* | _________________________________   \|  \|  \|  \|  \| \| --- \| --- \| --- \| --- \| |  |
| Q1032 | What is the highest level of education that he completed? | 1. *No formal education* 2. *Less than primary school* 3. *Primary school completed* 4. *Secondary school completed* 5. *High school (or equivalent) completed* 6. *College/pre-university completed* 7. *Post graduate degree completed*   *8 DK* |  |

Time End :

Thank you, that ends this section – we will return to questions about you in the next section.

Section 2000: Health State Descriptions

Time Begin :

Now we will switch to questions specifically about your health. The first questions are about your overall health, including both your physical and your mental health.

| Q2000 | In general, how would you rate your health today? | 1. Very good 2. Good 3. Moderate 4. Bad 5. Very bad |
| --- | --- | --- |
| Q2001 | Overall in the last 30 days, how much difficulty did you have with work or household activities? | 1. None 2. Mild 3. Moderate 4. Severe 5. Extreme/cannot do |

Now I would like to review the different functions of your body. When answering these questions, I would like you to think about the last 30 days, taking both good and bad days into account. When I ask about difficulty, I would like you to consider how much difficulty you have had, on average, in the last 30 days, while doing the activity in the way that you usually do it. By difficulty I mean requiring increased effort, discomfort or pain, slowness or changes in the way you do the activity.

*INTERVIEWER: Read and show scale to respondent*

Mobility

|  | Overall in the last 30 days, how much difficulty did you have … | None | Mild | Moderate | Severe | Extreme / Cannot do |
| --- | --- | --- | --- | --- | --- | --- |
| Q2002 | … with moving around? | *1* | *2* | *3* | *4* | *5* |
| Q2003 | … in vigorous activities ('vigorous activities' require hard physical effort and cause large increases in breathing or heart rate)? | *1* | *2* | *3* | *4* | *5* |

Self-Care

|  | Overall in the last 30 days, how much difficulty did you have … | *None* | *Mild* | *Moderate* | *Severe* | *Extreme / Cannot do* |
| --- | --- | --- | --- | --- | --- | --- |
| Q2004 | … with self-care, such as bathing/washing or dressing yourself? | *1* | *2* | *3* | *4* | *5* |
| Q2005 | … in taking care of and maintaining your general appearance (for example, grooming, looking neat and tidy)? | *1* | *2* | *3* | *4* | *5* |
| Q2006 | … in staying by yourself for a few days (3 to 7 days)? | *1* | *2* | *3* | *4* | *5* |

Pain and Discomfort

|  | Overall in the last 30 days,… | *None* | *Mild* | *Moderate* | *Severe* | *Extreme / Cannot do* |
| --- | --- | --- | --- | --- | --- | --- |
| Q2007 | …how much of bodily aches or pains did you have? | *1* | *2* | *3* | *4* | *5* |
| Q2008 | …how much bodily discomfort did you have? | *1* | *2* | *3* | *4* | *5* |
| If Q2007 and Q2008 are both = 1, "None"…………………………………………………………..….🡺 | | | | | | *Q2010* |
| Q2009 | … how much difficulty did you have in your daily life because of your pain? | *1* | *2* | *3* | *4* | *5* |

Cognition

|  | Overall in the last 30 days, how much difficulty… | *None* | *Mild* | *Moderate* | *Severe* | *Extreme / Cannot do* |
| --- | --- | --- | --- | --- | --- | --- |
| Q2010 | … did you have with concentrating or remembering things? | *1* | *2* | *3* | *4* | *5* |
| Q2011 | … did you have in learning a new task (for example, learning how to get to a new place, learning a new game, learning a new recipe)? | *1* | *2* | *3* | *4* | *5* |

Interpersonal Activities

|  | Overall in the last 30 days, how much difficulty did you have,… | *None* | *Mild* | *Moderate* | *Severe* | *Extreme / Cannot do* |
| --- | --- | --- | --- | --- | --- | --- |
| Q2012 | … with personal relationships or participation in the community? | *1* | *2* | *3* | *4* | *5* |
| Q2013 | … in dealing with conflicts and tensions with others? | *1* | *2* | *3* | *4* | *5* |
| Q2014 | … with making new friendships or maintaining current friendships? | *1* | *2* | *3* | *4* | *5* |
| Q2015 | …with dealing with strangers? | *1* | *2* | *3* | *4* | *5* |

Sleep and Energy

|  | Overall in the last 30 days, how much of a problem did you… | *None* | *Mild* | *Moderate* | *Severe* | *Extreme / Cannot do* |
| --- | --- | --- | --- | --- | --- | --- |
| Q2016 | … have with sleeping, such as falling asleep, waking up frequently during the night or waking up too early in the morning? | *1* | *2* | *3* | *4* | *5* |
| Q2017 | … have due to not feeling rested and refreshed during the day (for example, feeling tired, not having energy)? | *1* | *2* | *3* | *4* | *5* |

Affect

|  | Overall in the last 30 days, how much of a problem did you have… | *None* | *Mild* | *Moderate* | *Severe* | *Extreme / Cannot do* |
| --- | --- | --- | --- | --- | --- | --- |
| Q2018 | …with feeling sad, low or depressed? | *1* | *2* | *3* | *4* | *5* |
| Q2019 | … with worry or anxiety? | *1* | *2* | *3* | *4* | *5* |

Vision *(Respondent should answer, as when wearing glasses/contact lenses if used)*

| Q2020 | When was the last time you had your eyes examined by a medical professional?  *Interviewer: enter years or months ago. Enter "00" if less than 1 year.* | *years ago*  *-8 DK*  *98 Never* |
| --- | --- | --- |
| Q2021 | Do you use eyeglasses or contact lenses to see far away (for example, across the street)? | 1. *Yes* 2. *No* |
| Q2022 | Do you use eyeglasses or contact lenses to see up close (for example, at arm’s length, like when you are reading)? | 1. *Yes* 2. *No* |
| Q2023 | In the last 30 days, how much difficulty did you have in seeing and recognising an object or a person you know across the road (from a distance of about 20 meters)? | 1. None 2. Mild 3. Moderate 4. Severe 5. Extreme / cannot do |
| Q2024 | In the last 30 days, how much difficulty did you have in seeing and recognising an object at arm's length (for example, reading)? | 1. *None* 2. *Mild* 3. *Moderate* 4. *Severe* 5. *Extreme / cannot do* |

Hearing *(respondent should answer as when wearing hearing aid if one is used)*

| Q2050 | Do you wear a hearing aid? | 1. Yes 2. No |
| --- | --- | --- |
| Q2051 | In the last 30 days, how much difficulty did you have in: hearing someone talking on the other side of the room in a normal voice (even with your hearing aid on if you use one)? | 1. None 2. Mild 3. Moderate 4. Severe 5. Extreme/cannot do |
| Q2052 | In the last 30 days, how much difficulty did you have in hearing what is said in a conversation with one other person in a quiet room (even with your hearing aid on if you use one)? | 1. None 2. Mild 3. Moderate 4. Severe 5. Extreme/cannot do |

##### Functioning assessment

These next questions ask about difficulties due to health conditions. Health conditions include diseases or illnesses, other health problems that may be short or long lasting, injuries, mental or emotional problems, and problems with alcohol or drugs.

Think back over the last 30 days and answer these questions thinking about how much difficulty you had doing the following activities. Some of these questions may seem repetitive, but we do need your attention and it is important to give us answers to each question.

Interviewer: For each question, please circle only one response.

|  | In the last 30 days, how much difficulty did you have … | None | Mild | Moderate | Severe | Extreme/  cannot do | *N/A* |
| --- | --- | --- | --- | --- | --- | --- | --- |
| Q2028 | … in standing for long periods? | *1* | *2* | *3* | *4* | *5* | *9* |
| Q2032 | … in taking care of your household responsibilities? | *1* | *2* | *3* | *4* | *5* | *9* |
| Q2033 | … in joining in community activities (for example, festivities, religious or other activities) in the same way as anyone else can? | *1* | *2* | *3* | *4* | *5* | *9* |
| Q2035 | … concentrating on doing something for 10 minutes? | *1* | *2* | *3* | *4* | *5* | *9* |
| Q2036 | … in walking a long distance such as a kilometer? | *1* | *2* | *3* | *4* | *5* | *9* |
| Q2037 | … in bathing/washing your whole body? | *1* | *2* | *3* | *4* | *5* | *9* |
| Q2038 | … in getting dressed? | *1* | *2* | *3* | *4* | *5* | *9* |
| Q2039 | … in your day to day work? | *1* | *2* | *3* | *4* | *5* | *9* |
| Q2040 | … with carrying things? | *1* | *2* | *3* | *4* | *5* | *9* |
| Q2042 | … with eating (including cutting up your food)? | *1* | *2* | *3* | *4* | *5* | *9* |
| Q2043 | … with getting up from lying down? | *1* | *2* | *3* | *4* | *5* | *9* |
| Q2044 | … with getting to and using the toilet? | *1* | *2* | *3* | *4* | *5* | *9* |
| Q2044a | …with control of your bowel or bladder functions? | *1* | *2* | *3* | *4* | *5* | *9* |
| Q2045 | … with getting where you want to go, using private or public transport if needed? | *1* | *2* | *3* | *4* | *5* | *9* |
| Q2046 | … getting out of your home? | *1* | *2* | *3* | *4* | *5* | *9* |
| Q2047 | In the last 30 days, how much have you been emotionally affected by your health condition(s)? | *1* | *2* | *3* | *4* | *5* | *9* |

| Q2053 | Overall, in the past 30 days, on how many days were these difficulties present? | days  *-8 DK*  *98 Never* |
| --- | --- | --- |

I would like to end this section with a question about your health when you were a child – think about your childhood, particularly before the age of 10. I would like to know about your health overall when you were younger than 10 years old.

| Q2054 | In general, how would you rate your health when you were a child, before the age of 10? | 1. Very good 2. Good 3. Moderate 4. Bad 5. Very bad |
| --- | --- | --- |

Time End :

# **Section 2500: Anthropometrics, Performance Tests and Biomarkers**

| Time Begin :  Before we ask you more questions, this time about your own health and well-being, we would like to measure a few things, like your blood pressure, your weight and height. We'll also ask you to participate in a few tests to determine your health status. |
| --- |

BloodPressure

| First I would like to measure your blood pressure and pulse rate. Stay seated, and once I put this on your wrist, keep it steady and at the level of your heart. We will need to take the blood pressure reading three times. It will squeeze your wrist a bit, but won't hurt. Relax.  *INTERVIEWER: respondent should remain seated. Demonstrate to the respondent how to hold their arm while the machine is measuring. Place the monitoring device on the wrist and have the respondent hold it at heart level against his/her chest. When the device is in the correct position and respondent is relaxed, press the button to start. Check to make sure it is working. Collect the blood pressure and pulse 3 times with one minute between each measurement. You do not need to remove the device between measurements.* | |
| --- | --- |
| Q2501 | *Time 1 Systolic*  *Diastolic* |
| Q2501a | *Time 1 Pulse rate / minute* |
| *INTERVIEWER: Ask the respondent to release the arm and relax. Wait for one minute before time 2.*  Okay, now we can get your second measurement for your blood pressure. | |
| Q2502 | *Time 2 Systolic*  *Diastolic* |
| Q2502a | *Time 2 Pulse rate / minute* |
| *INTERVIEWER: Again, remind the respondent to relax. Meanwhile, when waiting to take the third measurement, you can locate and measure out a 4 metre length to prepare for the vision test and timed walk.*  *Okay, now we can get your third measurement for your blood pressure.* | |
| Q2503 | *Time 3 Systolic*  *Diastolic* |
| Q2503a | *Time 3 Pulse rate*  */ minute* |
|  | *Notes:* |

| *FILTER1* | *INTERVIEWER: Can respondent stand up, yes or no?* | 1. *Yes* 2. *No …………………… 🡺* | *Q2514* |
| --- | --- | --- | --- |

Anthropometric Measurements

| *I would now like to measure how tall you are. To measure your height I need you to please take off your shoes. Put your feet and heels close together, stand straight and look forward standing with your back, head and heels touching the wall. Look straight ahead.* | | |
| --- | --- | --- |
| *Q2506* | *Measured height*  *in centimetres* | . *centimetres*  *997 Refused*  *998 Not able* |
| *Now we want to measure your weight - could you please keep your shoes off and step on this scale. We will also measure your waist and hips using a tape measure.* | | |
| *Q2507* | *Measured weight*  *In kilograms* | . *kilograms*  *997 Refused*  *998 Not able* |
| *Q2508* | *Waist circumference*  *INTERVIEWER: identify the top of the hip bone - and make sure the tape measure is parallel to the floor all the way around the body* | . *centimetres*  *997 Refused*  *998 Not able* |
| *Q2509* | *Hip circumference*  *INTERVIEWER: measure at the maximum circumference of the hips - and make sure the tape measure is parallel to the floor all the way around the body* | . *centimetres*  *997 Refused*  *998 Not able* |
| *Now you can put your shoes back on, if you wish, and we can continue.* | | |
| *Notes:* | | |

Timed Walk

*INTERVIEWER: you will now invite the respondent to do a walking test - using your flexible steel tape measure, mark out length of 4 metres over a flat and straight surface if you have not already done so. Mark the ground at the beginning and end. Mark sure the surface is flat and free of obstacles. You will walk slightly behind the person for both tests.*

Normal walk

Now I am going to observe how you normally walk. If you use a cane or other walking aid and would be more

comfortable with it, then you may use it. This is the walking course. I want you to walk to the other end of the course at your usual speed, just as if you were walking down the street to go to the store. Walk all the way past the other end of the tape before you stop. I will walk with you.

*INTERVIEWER: DEMONSTRATE.*

Do you feel this would be safe? *If yes, continue.*

When I want you to start, I will say: “Ready, begin.”

Ready begin.

| *Q2510* | *Did respondent complete the walk at usual pace?* | 1. *Yes* 2. *No, refused* 3. *No, cannot walk, even with support ………… 🡺* | *Q2514* |
| --- | --- | --- | --- |
| *Q2511* | *Time at 4 metres* | . *Seconds* | |

Rapid walk

Now I want to repeat the walk. This time, however, I would like you to walk at a rapid pace, as fast as you safely can, and go all the way past the other end of the course I marked out for you.

*INTERVIEWER: DEMONSTRATE.*

When I want you to start, I will say: “Ready, begin.”

“Ready begin.”

| *Q2512* | *Did respondent complete the walk at rapid pace?* | 1. *Yes* 2. *No, refused/unable …..🡺* | *Q2514* |
| --- | --- | --- | --- |
| *Q2513* | *Time at 4 metres* | . *Seconds* | |

Vision Test

We are now going to test your distance vision and near vision.

*INTERVIEWER: Invite the respondent to sit again - in a chair positioned so that the respondent's head will be 3 meters from the vision chart. Make sure the person does not lean in closer to the chart during the test.*

*To measure acuity in the left eye, the right eye is covered with right palm or an eye patch and the subject is asked to respond to each "E" that appears on the chart. When the subject has difficulty, s/he is encouraged to guess. Responses can be verbal (Up, Down, Left, Right) or the respondent can indicate with a finger.*

*The right eye can then be tested in the same way.*

Distance vision

*INTERVIEWER: Start with the distance vision.*

We will start with your distance vision - and with your left eye. Would you please cover your right eye with the palm of your right hand. Please read ….

| *Q2514* | *Distance Vision - Left Eye* | 1. 6/18 | 2. 6/60 | 3. 3/60 |
| --- | --- | --- | --- | --- |

Now cover your left eye with left hand so we can test your right eye.

Please read….

| *Q2515* | *Distance Vision - Right Eye* | 1. 6/18 | 2. 6/60 | 3. 3/60 |
| --- | --- | --- | --- | --- |

Near Vision

*INTERVIEWER: Responses will be verbal (Up, Down, Left, Right).*

Okay, now we would like to test your near vision - starting again with your left eye - please cover your right eye with your right hand. Indicate if the "E" is facing Up, Down, Left or Right. Please read….

| *Q2516* | *Near Vision - Left Eye* | 1. N48 | 2. N20 | 3. N8 |
| --- | --- | --- | --- | --- |

Now cover your left eye with left hand so we can test your right eye.

Please read….

| *Q2517* | *Near Vision - Right Eye* | 1. N48 | 2. N20 | 3. N8 |
| --- | --- | --- | --- | --- |

Grip strength

*FILTER: If respondent has obvious problem with hand/arm, skip that side. If problems with both hands/arms, answer 1=yes to Q2518 and Q2519, then* 🡺 *skip to Q2525. Make sure you fit the dynamometer to the respondent's hand size.*

We are now going to test the strength in your hands.

| Q2518 | *Have you had any surgery on your left arm, hand or wrist in the last 3 months OR arthritis or pain in your left hand or wrist?* | 1. *Yes 🡺 Do not test Left hand* 2. *No* |
| --- | --- | --- |
| Q2519 | *Have you had any surgery on your right arm, hand or wrist in the last 3 months OR arthritis or pain in your right hand or wrist?* | 1. *Yes 🡺 Do not test Right hand* 2. *No* |
| Q2520 | *Which hand do you consider your dominant hand?* | 1. *Left* 2. *Right* 3. *Use both the same* |

Remain sitting and let your hand drop to your side. Keep your upper arm against your body and bend your elbow to 90 degrees with palm facing in (like shaking hands). Keep your elbow pressed against your side. *INTERVIEWER: DEMONSTRATE.*

Then grab the two pieces of metal together like this.

*INTERVIEWER: DEMONSTRATE.*

I will ask you to do this two times in each hand. Let’s start with your left hand, please take this in your left hand.

If you feel any pain or discomfort, tell me and we will stop.

When I say "squeeze", squeeze as hard as you can.

*Interviewer: Check positioning and grip to make sure it is correct. When he or she begins, say: Squeeze, squeeze, squeeze!*

Ready? Squeeze, squeeze, squeeze!

| Q2521 | *First test left hand* | *kilograms*  *-9 Refused ………………🡺* | *Q2523* |
| --- | --- | --- | --- |
| Q2522 | *Second test left hand* | *kilograms* | |

Okay, now let’s do the same on the other side. Hold the device in your right hand, so we can test your strength on this side also.

*INTERVIEWER: Check positioning and grip to make sure it is correct.*

Ready? Squeeze, squeeze, squeeze!

| *Q2523* | *First test right hand* | *kilograms*  *-9 Refused ………………🡺* | *Q2525* |
| --- | --- | --- | --- |
| *Q2524* | *Second test right hand* | *kilograms* | |

Verbal Recall

We are now going to test your memory. I know these questions may be difficult to answer, but please try to provide an answer. I am going to read you a list of words. Listen to them carefully and try to remember as many of them as you can, not necessarily in order. I will ask you to repeat them again after some time.

*INTERVIEWER: you can use the table below to assist you with scoring. Press START/STOP on stopwatch. Time for one minute for each trial.*

| *LIST OF WORDS:* | | *Trial 1* | *Trial 2* | *Trial 3* |
| --- | --- | --- | --- | --- |
| *Arm* | |  |  |  |
| *Bed* | |  |  |  |
| *Plane* | |  |  |  |
| *Dog* | |  |  |  |
| *Clock* | |  |  |  |
| *Bike* | |  |  |  |
| *Ear* | |  |  |  |
| *Hammer* | |  |  |  |
| *Chair* | |  |  |  |
| *Cat* | |  |  |  |
| *Substituted words:* | |  |  |  |
| Q2525 | *Number of words recalled correctly Trial 1* |  |  |  |
| Q2526 | *Number of words that respondent failed to recall Trial 1* |  |  |  |
| Q2527 | *Number of words substituted Trial 1* |  |  |  |
| *I will read the list to you again, and then again when I am done, repeat them after me.* | | | | |
| Q2528 | *Number of words recalled correctly Trial 2* |  |  |  |
| Q2529 | *Number of words that respondent failed to recall Trial 2* |  |  |  |
| Q2530 | *Number of words substituted Trial 2* |  |  |  |
| One final time - I will read the list and when I am done, you repeat as many as you can remember. | | | | |
| Q2531 | *Number of words recalled correctly Trial 3* |  |  |  |
| Q2532 | *Number of words that respondent failed to recall Trial 3* |  |  |  |
| Q2533 | *Number of words substituted Trial 3* |  |  |  |

Digit span - Digits forward

*INTERVIEWER: For the following tests, digits forward and backward, say the digits at the rate of one per*

*second, not grouped. Let the pitch of your voice drop with the last digit of each series. In any series if the subject fails Trial 1 - give Trial 2 of the same series, then proceed to the next series if the respondent responds correctly. Trial 2 is only given if Trial 1 is failed.*

I am going to say some numbers to you. Listen carefully, and when I am through, say them right after me. I want you to repeat each set of numbers exactly as I say them to you. For example, if I said "1-2", you would say…?

*INTERVIEWER: Wait for correct response "1-2". If correct, start with Series 3. If response is incorrect, provide the correct response and attempt once more with another example.*  Okay, let's try another example, repeat after me, "5-3".  *If correct, continue. If not correct - mark "0" in Q2534 and* 🡺 *skip to Verbal Fluency (Q2536).*

**Okay, good. Let us start with the numbers.**

*INTERVIEWER: Stop when respondent fails both trials.*

| Series | Trial 1 | Trial 1 Correct? | Trial 2 | Trial 2 Correct? |
| --- | --- | --- | --- | --- |
| 3 | 5-8-2 | *Yes 🡺 Series 4*  *No 🡺 Trial 2* | 6-9-4 | *Yes 🡺 Series 4*  *No 🡺 End* |
| 4 | 6-4-3-9 | *Yes 🡺 Series 5*  *No 🡺 Trial 2* | 7-2-8-6 | *Yes 🡺 Series 5*  *No 🡺 End* |
| 5 | 4-2-7-3-1 | *Yes 🡺* Series *6*  *No 🡺 Trial 2* | 7-5-8-3-6 | *Yes 🡺 Series 6*  *No 🡺End* |
| 6 | 6-1-9-4-7-3 | *Yes 🡺* Series *7*  *No 🡺 Trial 2* | 3-9-2-4-8-7 | *Yes 🡺 Series 7*  *No 🡺 End* |
| 7 | 5-9-1-7-4-2-8 | *Yes 🡺* Series *8*  *No 🡺 Trial 2* | 4-1-7-9-3-8-6 | *Yes 🡺 Series 8*  *No 🡺 End* |
| 8 | 5-8-1-9-2-6-4-7 | *Yes 🡺* Series *9*  *No 🡺 Trial 2* | 3-8-2-9-5-1-7-4 | *Yes 🡺 Series 9*  *No 🡺 End* |
| 9 | 2-7-5-8-6-2-5-8-4 | *Yes 🡺 end*  *No 🡺 Trial 2* | 7-1-3-9-4-2-5-6-8 | *Yes 🡺 end*  *No 🡺 End* |

Okay good.  *INTERVIEWER: mark the score in Q2534*

| *Q2534* | *Total score (the series number in the longest series repeated without error in Trial 1 or 2)*  *(Maximum = 9 points)* |  |
| --- | --- | --- |

Digit span - Digits backward

Now, I am going to say more numbers, but this time when I stop, I want you to say them to me backwards.

For example, if I said 1-7, what would you say?

*INTERVIEWER: Wait for subject to say 7-1. If response is correct, start with Series 2. If respondent does not reply correctly or fails to understand, give the correct answer and another example, saying* Remember, you are to say them backwards. Try this, "3-8". *If response is correct, continue. If fails second example, mark "0" in Q2535 and skip to Verbal Fluency (Q2536).*

**Okay, lets start.**

| Series | Trial 1 | Trial 1 Correct? | Trial 2 | Trial 2 Correct? |
| --- | --- | --- | --- | --- |
| 2 | 2-4 | *Yes 🡺 Series 3*  *No 🡺 Trial 2* | 5-8 | *Yes 🡺 Series 3*  *No 🡺 End* |
| 3 | 6-2-9 | *Yes 🡺 Series 4*  *No 🡺 Trial 2* | 4-1-5 | *Yes 🡺 Series 4*  *No 🡺 End* |
| 4 | 3-2-7-9 | *Yes 🡺 Series 5*  *No 🡺 Trial 2* | 4-9-6-8 | *Yes 🡺 Series 5*  *No 🡺End* |
| 5 | 1-5-2-8-6 | *Yes 🡺 Series 6*  *No 🡺 Trial 2* | 6-1-8-4-3 | *Yes 🡺 Series 6*  *No 🡺 End* |
| 6 | 5-3-9-4-1-8 | *Yes 🡺 Series 7*  *No 🡺 Trial 2* | 7-2-4-8-5-6 | *Yes 🡺 Series 7*  *No 🡺 End* |
| 7 | 8-1-2-9-3-6-5 | *Yes 🡺 Series 8*  *No 🡺 Trial 2* | 4-7-3-9-1-2-8 | *Yes 🡺 Series 8*  *No 🡺 End* |
| 8 | 9-4-3-7-6-2-5-8 | *Yes 🡺 end*  *No 🡺 Trial 2* | 7-2-8-1-9-6-5-3 | *Yes 🡺 end*  *No 🡺 End* |

Okay, good. *INTERVIEWER: mark score in Q2535.*

| *Q2535* | *Total score (the series number in the longest series repeated without error in Trial 1 or 2)*  *(Maximum = 8 points)* |  |
| --- | --- | --- |

Verbal Fluency

Now we are going to ask you to think of animals and name as many as you can. I am going to give you one minute and I want to see how many animals you can name.

*INTERVIEWER: See Interviewers Manual instructions about what is acceptable and what is not. If respondent stops before the end of the minute, encourage them to try to name more animals. If there is a silence of about 15 seconds, prompt them to continue or repeat the basic instructions.*

Ready? Start:

*INTERVIEWER: Press START/STOP on stopwatch. Time for one minute. Use space below to record.*

*INTERVIEWER: SAY “FINE” OR “GOOD” when completed the one minute.*

| *Q2536* | *Total score (number of animals named correctly)* |  |
| --- | --- | --- |
| *Q2537* | *Number of errors*  *INTERVIEWER: errors include anything that is not an animal* |  |

Delayed Verbal Recall

I read you a list of words about 10 minutes ago. I will NOT repeat this list to you now, but could you please repeat to me as many of them as you can remember?

*INTERVIEWER - DO NOT read the list again to the respondent - the list below is for your own use.*

| *LIST OF WORDS:* | | |  |  |
| --- | --- | --- | --- | --- |
| *Arm* | | *Bike* |  |  |
| *Bed* | | *Ear* |  |  |
| *Plane* | | *Hammer* |  |  |
| *Dog* | | *Chair* |  |  |
| *Clock* | | *Cat* |  |  |
| *Q2544* | *Number of words recalled correctly* | | |  |
| *Q2545* | *Number of words that respondent failed to recall* | | |  |
| *Q2546* | *Number of words substituted* | | |  |

Time End :

That is the end of this section, now we will move onto questions about your health behaviours.

Section 3000: Risk Factors and Preventive Health Behaviours

Time Begin :

We would now like to ask you some questions about your habits, health behaviours and awareness about health. This includes things like smoking, drinking alcohol, eating enough fruits and vegetables as part of your diet and your levels of physical activity. I will start with questions about smoking habits.

Tobacco use *(see appendix A3000A)*

| Q3001 | Have you ever smoked tobacco or used smokeless tobacco? | 1. Yes 2. No ………………………….🡺 | Q3007 |
| --- | --- | --- | --- |
| Q3002 | Do you **currently use (smoke, sniff or chew)** any tobacco products (such as cigarettes, bidis, cigars, pipes, chewing tobacco or snuff)? | 1. Yes, daily 2. Yes, but not daily 3. No, not at all …………..🡺 | Q3005 |
| Q3003 | For how long have you been smoking tobacco?  *INTERVIEWER: If less than one month – enter “00” for years and "00" for months.* | Years Months  *-8 DK* |  |

| Q3004 | *INTERVIEWER: If Q3002=1 use “each day”, if Q3002=2 use “each week”.*  On average, how many of the following products do you smoke or use each day/week? Also, let me know if you smoke the product, but not every (day/week). | *Include number below:*  *INTERVIEWER: If respondent reports smoking the product, but not every day/week, enter 888* |  |
| --- | --- | --- | --- |

|  | Q3004a. Manufactured cigarettes |  |  |
| --- | --- | --- | --- |
|  | Q3004b. Hand-rolled cigarettes |  |  |
|  | Q3004c. Pipefuls of tobacco |  |  |
|  | Q3004d. Cigars, cheroots, cigarillos, |  |  |
|  | Q3004e. Bidis |  |  |
|  | Q3004f. Other, specify: | ……..*…………………....*.🡺 | If Q3002 = 1 -🡪 Q3007  If Q3002 = 2 -🡪 Q3005a |
| Q3005 | In the past, did you ever smoke tobacco?  *INTERVIEWER: If respondent has done both daily and less than daily in the past, check: 1 Yes, daily.* | 1. Yes, daily …………..*……….....*.🡺 2. Yes, but not daily …………..*....*.🡺 3. No …………..*……………….....*.🡺 | Q3006  Q3006  Q3007 |
| Q3005a | Have you smoked tobacco daily in the past? | 1. Yes…………..*…………………….....*.🡺 2. No…………..*……………………......*.🡺 | Q3007  Q3007 |
| Q3006 | How old were you when you stopped smoking tobacco? | Years of age *……...*.🡺  -8 *DK …….……………...*.🡺 | Q3007  Q3006a |
|  | **Q3006**a**.** How long ago did you stop smoking tobacco?  *INTERVIEWER: If less than one month – enter “00” for years and “00” for months.* | Years ago Months ago  -8 *DK* | |

Alcohol *(show Alcohol card to respondent - see Appendix A3000B)*

| *Q3007* | Have you ever consumed a drink that contains alcohol (such as beer, wine, spirits)? | 1. *Yes* 2. *No, Never ………………….….🡺* | *Q3012* |
| --- | --- | --- | --- |
| *Q3007a* | How old were you when you first started consuming alcohol? |  |  |
| *Q3007b* | How often do you have a drink containing alcohol? | 0 No days *…*………………….. 🡺   1. Monthly or less 2. 2-4 times a month 3. 2-3 times a week   4 4 or more times a week | *Q3008* |
| *Q3007c* | How many standard drinks containing alcohol do you have on a typical day? | 1. 1 or 2 2. 3 or 4 3. 5 or 6 4. 7 to 9   5 10 or more |  |
| *Q3008* | Have you consumed alcohol in the last 30 days? | 1. *Yes* 2. *No* *………………………..…….🡺* | *Q3010* |
| Q3009 | During the past 7 days, how many drinks of any alcoholic beverage did you have each day?  *USE SHOWCARD Appendix A3000B.* | *INTERVIEWER: Want respondent to tell you the number of "standard" drinks. By standard drink - refer to Appendix. Include number below:* | |
|  | *Q3009a. Monday* |  | |
|  | *Q3009b. Tuesday* |  | |
|  | *Q3009c. Wednesday* |  | |
|  | *Q3009d. Thursday* |  | |
|  | *Q3009e. Friday* |  | |
|  | *Q3009f. Saturday* |  | |
|  | *Q3009g. Sunday* |  | |
| Q3010 | In the last 12 months, how frequently [on how many days] on average, have you had at least one alcoholic drink? | *0 No days …*…………………..…….….🡺   1. *Less than once a month* 2. *One to three days per month* 3. *One to four days per week* 4. *Five or more days per week* | *Q3012* |
| Q3011 | In the last 12 months, on the days you drank alcoholic beverages, how many drinks did you have on average? | *Drinks*  *-8 DK* | |

Nutrition

Studies have shown that nutrition and life-style are very important health factors. I want to ask you a few questions about your diet. I am going to ask you about the fruit and vegetables you usually eat.

*(Show Nutrition card to respondent -- see Appendix A3000C)*

| Q3012 | How many servings of fruit do you eat on a typical day? | Servings  -8 DK | |
| --- | --- | --- | --- |
| Q3013 | How many servings of vegetables do you eat on a typical day? | Servings  -8 DK | |
| Q3015a | Do you add salt to food at the table? | 1. Always 2. Rarely 3. Sometimes 4. Often 5. Never | |
| Q3015b | In the food you eat at home, salt is added in cooking…? | 1. Always 2. Rarely 3. Sometimes 4. Often 5. Never | |
| Q3015c | How much salt do you think you consume?  *INTERVIEWER: READ LIST* | 1. Far too much 2. Too much 3. Just the right amount 4. Too little 5. Far too little   8 DK  9 Refused | |
| Q3015d | Do you think that a high salt diet could cause a serious health problem? | 1. Yes 2. No   8 DK  9 Refused |  |
| Q3015e | Do you do anything on a regular basis to control your salt or sodium intake? | 1. Yes 2. No   8 DK  9 Refused |  |
| Q3014 | In the last 12 months, how often did you ever eat less than you felt you should because there wasn’t enough food? | 1. Every month 2. Almost every month 3. Some months, but not every month 4. Only in 1 or 2 months 5. Never | |
| Q3015 | In the last 12 months, were you ever hungry, but didn’t eat because you couldn’t afford enough food? | 1. Every month 2. Almost every month 3. Some months, but not every month 4. Only in 1 or 2 months 5. Never | |

Physical Activity - *(see appendix A3000D)*

Next I am going to ask you about the time you spend doing different types of physical activity in a typical week. Please answer these questions even if you do not consider yourself to be an active person. Think first about the time you spend doing work. Think of work as the things that you have to do such as paid or unpaid work, household chores, harvesting food/crops, fishing or hunting for food, providing care or seeking employment.

In answering the following questions 'vigorous activities' require hard physical effort and cause large increases in breathing or heart rate, 'moderate activities' require moderate physical effort and cause small increases in breathing or heart rate.

| Q3016 | Does your work involve vigorous-intensity activity that causes large increases in breathing or heart rate, [like heavy lifting, digging or chopping wood] for at least 10 minutes continuously?  INSERT EXAMPLES & USE SHOWCARD | | | 1. Yes 2. No …………………………….🡺 | | *Q3019* |
| --- | --- | --- | --- | --- | --- | --- |
| Q3017 | In a typical week, on how many days do you do vigorous-intensity activities as part of your work? | | | days | | |
| Q3018 | How much time do you spend doing vigorous-intensity activities at work on a typical day? | | | :  Hours:Minutes | | |
| Q3019 | Does your work involve moderate-intensity activity that causes small increases in breathing or heart rate [such as brisk walking, carrying light loads, cleaning, cooking, or washing clothes] for at least 10 minutes continuously?  INSERT EXAMPLES & USE SHOWCARD | | | 1. Yes 2. No …………………………..🡺 | | *Q3022* |
| Q3020 | In a typical week, on how many days do you do moderate-intensity activities as part of your work? | | | days | | |
| Q3021 | How much time do you spend doing moderate-intensity activities at work on a typical day? | | | :  Hours:Minutes | | |
| The next questions exclude the physical activities at work that you’ve already mentioned.  Now I would like to ask you about the usual way you travel to and from places. For example, getting to work, to shopping, to the market, to place of worship. [Insert other examples if needed] | | | | | | |
| Q3022 | | | Do you walk or use a bicycle (pedal cycle) for at least 10 minutes continuously to get to and from places? | | 1. *Yes* 2. *No ………………………....🡺* | *q3025* |
| Q3023 | | | In a typical week, on how many days do you walk or bicycle for at least 10 minutes continuously to get to and from places? | | days |  |
| Q3024 | | | How much time would you spend walking or bicycling for travel on a typical day? | | :  Hours:Minutes | |
| The next questions exclude the work and transport activities that you have already mentioned. Now I would like to ask you about sports, fitness, leisure and recreational activities [insert relevant terms] | | | | | | |
| Q3025 | | Do you do any vigorous intensity sports, fitness or recreational (leisure) activities that cause large increases in breathing or heart rate [like running or football], for at least 10 minutes continuously?  *INSERT EXAMPLES & USE SHOWCARD* | | | 1. *Yes* 2. *No ………………….………🡺* | *q3028* |
| Q3026 | | In a typical week, on how many days do you do vigorous intensity sports, fitness or recreational (leisure) activities? | | | *days* |  |
| Q3027 | | How much time do you spend doing vigorous intensity sports, fitness or recreational activities on a typical day? | | | :  *Hours:Minutes* | |
| Q3028 | | Do you do any moderate-intensity sports, fitness or recreational (leisure) activities that causes a small increase in breathing or heart rate [such as brisk walking, cycling or swimming] for at least 10 minutes at a time?  *INSERT EXAMPLES & USE SHOWCARD* | | | 1. *Yes* 2. *No …………………….…..🡺* | *Q3031* |
| Q3029 | | In a typical week, on how many days do you do moderate-intensity sports, fitness or recreational (leisure) activities? | | | days | |
| Q3030 | | How much time do you spend doing moderate intensity sports, fitness or recreational (leisure) activities on a typical day? | | | :  Hours:Minutes | |
| The following question is about sitting or reclining at work, at home, getting to and from places, or with friends including time spent [sitting at a desk, sitting with friends, travelling in car, bus, train, reading, playing cards or watching television], but do not include time spent sleeping. INSERT EXAMPLES & USE SHOWCARD | | | | | | |
| *Q3031* | | How much time do you usually spend sitting or reclining on a typical day? | | | :  Hours:Minutes | |

Time End :

Section 4000: Chronic Conditions and Health Services Coverage

Time Begin :

Now I would like to ask you questions about some health problems or health care needs that you may have experienced, and the treatment or medical care that you may have received.

*Arthritis*

| Q4001 | Has a health care professional/doctor ever told you that you have arthritis (a disease of the joints)? | 1. *Yes* 2. *No* *…………………..……….🡺* | *Q4003* |
| --- | --- | --- | --- |
| Q4001a | When were you diagnosed?  *INTERVIEWER: If don’t know then ask:*  How long ago were you diagnosed? OR  How long have you had…arthritis? | Years ago  Months ago  -8 *DK* |  |
| Q4002 | If yes,  Have you been taking medications or other treatment for it….. | |  |
|  | **Q4002a.** …during the last 2 weeks? | 1. *Yes* 2. *No* |  |
|  | **Q4002b** …during the last 12 months? | 1. *Yes* 2. *No* |  |
| Q4003 | During the last 12 months have you experienced, pain, aching, stiffness or swelling in or around the joints (like arms, hands, legs or feet) which were not related to an injury and lasted for more than a month? | 1. *Yes* 2. *No* |  |
| Q4004 | During the last 12 months have you experienced, stiffness in the joint in the morning after getting up from bed, or after a long rest of the joint without movement? | 1. *Yes* 2. *No* *………………………….🡺* | *Q4007* |
| *If Q4003 and Q4004 are both "No" (that is, no symptoms of arthritis), skip to ……………………………….🡺* | | | *Q4008* |
| Q4005 | How long did this stiffness last? | 1. *About 30 minutes or less* 2. *More than 30 minutes* |  |
| Q4006 | Did this stiffness go away after exercise or movement in the joint? | 1. *Yes* 2. *No* |  |
| Q4007 | These symptoms that you have said you experienced in the last 12 months, have you experienced them in the last 2 weeks? | 1. *Yes* 2. *No* |  |
| Q4008 | Have you experienced back pain during the last 30 days? | 1. *Yes* 2. *No* *…………………….………..🡺* | *Q4010* |
| Q4009 | On how many days did you have this back pain during the last 30 days? | *Days* |  |

*Stroke*

| Q4010 | Has a health care professional/doctor ever told you that you have had a stroke? | 1. *Yes* 2. *No* *………………………..…….🡺* | *Q4012* |
| --- | --- | --- | --- |
| Q4010a | When were you diagnosed?  *INTERVIEWER: If don’t know then ask:*  How long ago were you diagnosed? OR  How long ago did you have a stroke/your last stroke? | Years ago Months ago  -8 *DK* |  |
| Q4011 | Have you been taking any medications or other treatment for it… | |  |
|  | **Q4011a.** …during the last 2 weeks? | 1. *Yes* 2. *No* |  |
|  | **Q4011b.** …during the last 12 months? | 1. *Yes* 2. *No* |  |
| Q4012 | Have you ever suffered from sudden onset of paralysis or weakness in your arms or legs on one side of your body for more than 24 hours? | 1. *Yes* 2. *No* |  |
| Q4013 | Have you ever had, for more than 24 hours, sudden onset of loss of feeling on one side of your body, without anything having happened to you immediately before? | 1. *Yes* 2. *No* |  |

*Angina*

| Q4014 | Has a health care professional/doctor ever told you that you have angina or angina pectoris (a heart disease)? | 1. *Yes* 2. *No ………………………..🡺* | *Q4016* |
| --- | --- | --- | --- |
| Q4014a | When were you diagnosed?  *INTERVIEWER: If don’t know then ask:*  How long ago were you diagnosed? OR  How long have you had…angina? | Years ago Months ago  -8 *DK* |  |
| Q4015 | Have you been taking any medications or other treatment for it… | |  |
|  | **Q4015a**...during the last 2 weeks? | 1. *Yes* 2. *No* |  |
|  | **Q4015b**...during the last 12 months? | 1. *Yes* 2. *No* |  |
| Q4016 | During the last 12 months, have you experienced any pain or discomfort in your chest when you walk uphill or hurry? | 1. *Yes* 2. *No* 3. *Never walks uphill or hurries* |  |
| Q4017 | During the last 12 months, have you experienced any pain or discomfort in your chest when you walk at an ordinary pace on level ground? | 1. *Yes* 2. *No*  *………………………….…🡺* | *Q4022* |
| Q4018 | What do you do if you get the pain or discomfort when you are walking?  *Read choices* | 1. Stop or slow down 2. Carry on after taking a pain relieving medicine that dissolves in your mouth 3. Carry on walking |  |
| Q4019 | If you stand still, what happens to the pain or discomfort?  *Read choices* | 1. Relieved 2. Not relieved |  |

| Q4020 | Will you show me where you usually experience the pain or discomfort?  *INTERVIEWER: Circle number in each of the boxes in the areas of body mentioned or shown by the respondent.* | *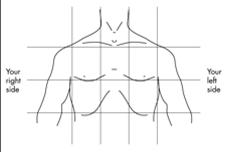*  17  18  16  15  14  13  12  10  11  9  8  7C  6  4  5  1  2  3 | | |
| --- | --- | --- | --- | --- |
| Q4021 | These symptoms that you have said you experienced in the last 12 months, have you experienced them in the last 2 weeks? | | 1. *Yes* 2. *No* |  |

*Diabetes*

| Q4022 | Have you ever been diagnosed with diabetes (high blood sugar)? | 1. *Yes* 2. *No* *……………………..🡺* | *Q4025* |
| --- | --- | --- | --- |
| Q4022a | When were you diagnosed?  *INTERVIEWER: If don’t know then ask:*  How long ago were you diagnosed? OR  How long have you had diabetes? | Years ago Months ago  -8 *DK* |  |
| Q4023 | Have you been taking insulin or other blood sugar lowering medications… | |  |
|  | **Q4023a** …in the last 2 weeks? | 1. *Yes* 2. *No* |  |
|  | **Q4023b** …in the last 12 months? | 1. *Yes* 2. *No* |  |
| Q4024 | Are you following a special diet, exercise regime or weight control program for diabetes during the last 2 weeks?  *(As recommended by health professional)* | 1. *Yes* 2. *No* |  |

*Chronic Lung Disease*

| Q4025 | Have you ever been told by a doctor or health care professional that you have chronic lung disease (emphysema, bronchitis, COPD)? | 1. *Yes* 2. *No* *……………………….🡺* | *Q4027* |
| --- | --- | --- | --- |
| *Q4025a* | When were you diagnosed?  *INTERVIEWER: If don’t know then ask:*  How long ago were you diagnosed? OR  How long have you had…COPD/emphysema? | Years ago Months ago  -8 *DK* |  |
| Q4026 | Have you been taking any medications or other treatment (like oxygen) for it … | |  |
|  | **Q4026a** …in the last 2 weeks? | 1. *Yes* 2. *No* |  |
|  | **Q4026b** …in the last 12 months? | 1. *Yes.* 2. *No* |  |
| Q4027 | During the last 12 months, have you experienced any shortness of breath at rest? (while awake) | 1. *Yes* 2. *No* |  |
| Q4028 | During the last 12 months, have you experienced any coughing or wheezing for ten minutes or more at a time? | 1. *Yes* 2. *No* |  |
| Q4029 | During the last 12 months, have you experienced any coughing up sputum or phlegm for most days of the month for at least 3 months? | 1. *Yes* 2. *No* |  |

*Asthma*

| Q4033 | Have you ever been diagnosed with asthma (an allergic respiratory disease)? | 1. *Yes* 2. *No* ……………………….…….🡺 | *Q4035* |
| --- | --- | --- | --- |
| Q4033a | When were you diagnosed?  *INTERVIEWER: If don’t know then ask:*  How long ago were you diagnosed? OR  How long have you had…asthma? | Years ago Months ago  -8 *DK* |  |
| Q4034 | Have you been taking any medications or other treatment for it … | |  |
|  | **Q4034a** …in the last 2 weeks? | 1. *Yes* 2. *No* |  |
|  | **Q4034b** …in the last 12 months? | 1. *Yes* 2. *No* |  |
| *During the last 12 months, have you experienced any of the following:* | | | |
| Q4035 | Attacks of wheezing or whistling breathing? | 1. *Yes* 2. *No* |  |
| Q4036 | Attack of wheezing that came on after you stopped exercising or some other physical activity? | 1. *Yes* 2. *No* |  |
| Q4037 | A feeling of tightness in your chest? | 1. *Yes* 2. *No* |  |
| Q4038 | Have you woken up with a feeling of tightness in your chest in the morning or any other time? | 1. *Yes* 2. *No* |  |
| Q4039 | Have you had an attack of shortness of breath that came on without obvious cause when you were not exercising or doing some physical activity? | 1. *Yes* 2. *No* |  |
| *IF Q4035 to Q4039 are all 'No', skip to ……………………………………………………………………………..🡺*  *If one of the symptom questions (Q4035 to Q4039) is 'Yes', continue with Q4039a.* | | | *Q4040* |
| Q4039a | These symptoms that you said you experienced in the last 12 months, have you experienced them in the last 2 weeks? | 1. *Yes* 2. *No* |  |

*Depression*

| Q4040 | Have you ever been told by a doctor that you have depression? | 1. *Yes* 2. *No* *…………………………..🡺* | *Q4042* |  |
| --- | --- | --- | --- | --- |
| Q4040a | When were you diagnosed?  INTERVIEWER: If don’t know then ask:  How long ago were you diagnosed? OR  How many years have you had depression? | Years ago Months ago  -8 *DK* |  |  |
| Q4041 | Have you been taking any medications or other treatment for it …  (Other treatment can include attending therapy or counseling sessions.) | |  |  |
|  | **Q4041a** …during the last 2 weeks? | 1. *Yes* 2. *No* |  |  |
|  | **Q4041b** …during the last 12 months? | 1. *Yes* 2. *No* |  |  |
| Q4042 | During the last 12 months, have you had a period lasting several days when you felt sad, empty or depressed? | 1. *Yes* 2. *No* |  |  |
| Q4043 | During the last 12 months, have you had a period lasting several days when you lost interest in most things you usually enjoy such as personal relationships, work or hobbies/recreation? | 1. *Yes* 2. *No* |  |  |
| Q4044 | During the last 12 months, have you had a period lasting several days when you have been feeling your energy decreased or that you are tired all the time? | 1. *Yes* 2. *No* |  |  |
| *Interviewer: If any one of Q4042, Q4043 or Q4044 is "Yes" , Continue to Q4045*  *If all 3 (Q4042, Q4043 AND Q4044) are "No" …🡺 Skip to Q4060* | | | | |
| Q4045 | Was this period [of sadness/loss of interest/low energy] for more than 2 weeks? | 1. *Yes* 2. *No*  *..*…………….………………🡺 | *Q4060* |  |
| Q4046 | Was this period [of sadness/loss of interest/low energy] most of the day, nearly every day? | 1. *Yes* 2. *No* |  |  |
| Q4047 | During this period, did you lose your appetite? | 1. *Yes* 2. *No* |  |  |
| Q4048 | Did you notice any slowing down in your thinking? | 1. *Yes* 2. *No* |  |  |
| Q4049 | Did you notice any problems falling asleep? | 1. *Yes* 2. *No* |  |  |
| Q4050 | Did you notice any problems waking up too early? | 1. *Yes* 2. *No* |  |  |
| Q4051 | During this period, did you have any difficulties concentrating; for example, listening to others, working, watching TV, listening to the radio? | 1. *Yes* 2. *No* |  |  |
| Q4052 | Did you notice any slowing down in your moving around? | 1. *Yes* 2. *No* |  |  |
| Q4053 | During this period, did you feel anxious and worried most days? | 1. *Yes* 2. *No* |  |  |
| Q4054 | During this period, were you so restless or jittery nearly every day that you paced up and down and couldn’t sit still? | 1. *Yes* 2. *No* |  |  |
| Q4055 | During this period, did you feel negative about yourself or like you had lost confidence? | 1. *Yes* 2. *No* |  |  |
| Q4056 | Did you frequently feel hopeless - that there was no way to improve things? | 1. *Yes* 2. *No* |  |  |
| Q4057 | During this period, did your interest in sex decrease? | 1. *Yes* 2. *No* |  |  |
| Q4058 | Did you think of death, or wish you were dead? | 1. *Yes* 2. *No* |  |  |
| Q4059 | During this period, did you ever try to end your life? | 1. *Yes* 2. *No* |  |  |

*Hypertension*

| Q4060 | Have you ever been told by a doctor or health care professional that you have high blood pressure (hypertension)? | 1. *Yes* 2. *No* *…………………….……….🡺* | *Q4062* |
| --- | --- | --- | --- |
| Q4060a | When were you diagnosed?  INTERVIEWER: If don’t know then ask:  How long ago were you diagnosed? OR  How many years have you had…hypertension? | Years ago Months ago  -8 *DK* |  |
| Q4061 | Have you been taking any medications or other treatment for it during …  (Other treatment might include weight loss programme or change in eating habits.) | |  |
|  | **Q4061a**....the last 2 weeks? | 1. *Yes* 2. *No* |  |
|  | **Q4061b**....the last 12 months? | 1. *Yes* 2. *No* |  |

*Cataracts*

| Q4062 | Have you ever been diagnosed with a cataract in one or both of your eyes (a cloudiness in the lens of the eye)? | 1. *Yes* 2. *No* *……………………….🡺*   *8 DK* | *Q4090* |
| --- | --- | --- | --- |
| Q4063 | In the last 5 years, have you had eye surgery to remove this cataract(s)? | 1. *Yes* 2. *No* |  |
| In the last 12 months have you experienced any of the following:… | | | |
| Q4064 | …cloudy or blurry vision? | 1. *Yes* 2. *No* | |
| Q4065 | …vision problems with light, such as glare from bright lights, or halos around lights? | 1. *Yes* 2. *No* | |

*Cancer*

| Q4090 | Have you ever been told by a doctor or health care professional that you have cancer? | | *1 Yes*  *2 No* *…………………….……….🡺* | *Q4066* |
| --- | --- | --- | --- | --- |
| Q4090a | When were you diagnosed?  INTERVIEWER: If don’t know then ask:  How long ago were you diagnosed? OR  How many years have you had…cancer? | | Years ago Months ago  -8 *DK* |  |
| Q4090b | What type of cancer? |  | |  |
| Q4091 | Have you been taking any medications or other treatment for it during …  (Other treatment might include surgery, radiation therapy, immunotherapy…) | | |  |
|  | **Q4091a**....the last 2 weeks? | | *1 Yes*  *2 No* |  |
|  | **Q4091b**....the last 12 months? | | *1 Yes*  *2 No* |  |

*Oral Health*

Now I would like you to tell me about the condition of your mouth and teeth.

| Q4066 | Have you lost all of your natural teeth? | 1. *Yes* 2. *No* |  |
| --- | --- | --- | --- |
| Q4067 | During the last 12 months, have you had any problems with your mouth and/or teeth (this includes problems with swallowing)? | 1. *Yes* 2. *No* *……………………….🡺* | *Q4069* |
| Q4068 | Have you received any treatment from a dentist or other oral health specialist during… | |  |
|  | **Q4068a** … the last 2 weeks? | 1. *Yes* 2. *No* |  |
|  | **Q4068b** … the last 12 months? | 1. *Yes* 2. *No* |  |

*Injuries*

| *Q4069* | In the last 12 months, have you been involved in a road traffic accident where you suffered from bodily injury? PROBE: This could have been an accident in which you were involved either as the occupant of a motor vehicle, or when you were riding a motorcycle or bicycle or walking. | 1. *Yes (if more than one accident, select the most recent to ask about in more detail below)* 2. *No* *……………………………..….🡺* | *Q4073* |
| --- | --- | --- | --- |
| *Q4070* | How did the injury happen? Was it an accident, did someone else do this to you, or did you do this to yourself? | 1. *It was an accident (unintentional)* 2. *Someone else did it to me deliberately (intentional)* 3. *I did it to myself deliberately (self-inflicted)*   *8 DK* | |

*Injuries continued…*

| Q4071 | Did you receive any medical care or treatment for your injuries? | 1. *Yes* 2. *No* |  |
| --- | --- | --- | --- |
| Q4072 | Did you suffer a physical disability as a result of being injured?  *INTERVIEWER: Disability is any restriction or lack of ability to perform an activity as before the injury.* | 1. *Yes* 2. *No ……………………….…..……..🡺* | *Q4073* |
|  | Q4072a. In what ways were you physically disabled?  *INTERVIEWER: Circle all that respondent selects.* | 1. *Unable to use hand or arm* 2. *Difficulty to use hand or arm* 3. *Walk with a limp* 4. *Loss of hearing* 5. *Loss of vision* 6. *Weakness or shortness of breath* 7. *Inability to remember things* 8. *Inability to chew*   *87 Other, specify:* | |
| Q4073 | In the last 12 months, have you had any other event where you suffered from bodily injury?  *INTERVIEWER: If more than one, ask respondent to think of the most recent event.* | 1. *Yes (if more than one event, select the most recent to ask about in more detail below)* 2. *No*  *………………………….……..🡺* | *Q4078* |
|  | **Q4073a.** Where were you when you were injured? | 1. *Home* 2. *School* 3. *Work*   *7 Other, specify :*  *8 DK* |  |
| Q4074 | What was the cause of this injury? | 1. *Fall* 2. *Struck/hit by person or object* 3. *Stabbed* 4. *Gun shot* 5. *Fire, flames or heat* 6. *Drowning or near-drowning* 7. *Poisoning* 8. *Animal bite* 9. *Electricity shock*   *87 Other, specify:*  *88 DK* |  |
| Q4075 | How did the injury happen? Was it an accident, did someone else do this to you, or did you do this to yourself? | 1. *It was an accident (unintentional)* 2. *Someone else did it to me deliberately (intentional)* 3. *I did it to myself deliberately (self-inflicted)*   *8 DK* | |
| Q4076 | Did you receive any medical care or treatment for your injuries? | 1. *Yes* 2. *No* |  |

*Injuries continued…*

| *Q4077* | Did you suffer a physical disability as a result of being injured?  *INTERVIEWER: disability is any restriction or lack of ability to perform an activity as before the injury.* | 1. *Yes* 2. *No …………………………..🡺* | *Q4078* |
| --- | --- | --- | --- |
|  | **Q4077a.** In what ways were you physically disabled?  *INTERVIEWER: Circle all that respondent selects.* | 1. *Unable to use hand or arm* 2. *Difficulty to use hand or arm* 3. *Walk with a limp* 4. *Loss of hearing* 5. *Loss of vision* 6. *Weakness or shortness of breath* 7. *Inability to remember things* 8. *Inability to chew*   *87 Other, specify:* | |

*Cervical cancer and breast cancer screening* (Women only)

*Questions to be asked to FEMALE respondents only.*

Female *…..*🡺 Q4078

Male …..*…..*🡺 GO TO NEXT SECTION

Now I would like to ask you about some of the kinds of medical care or tests that you may have received.

| Q4078 | When was the last time you had a pelvic examination, if ever?  *(By pelvic examination, I mean when a doctor or nurse examined your vagina and uterus?)*  *Enter "00" if less than 1 year ago.*  *FOLLOW-UP (same question, just remove the “…if ever?”)* | Years ago  *98 Never had exam* *……………..…....🡺* | *Q4080* |
| --- | --- | --- | --- |
| Q4079 | The last time you had the pelvic examination, did you have a PAP smear test?  *(By PAP smear test, I mean did a doctor or nurse use a swab or stick to wipe from inside your vagina, take a sample and send it to a laboratory?)* | 1. Yes 2. No |  |
| Q4080 | When was the last time you had a mammography, if ever?  *(That is, an x-ray of your breasts taken to detect breast cancer at an early stage.)*  *Enter "00" if less than 1 year ago.*  *FOLLOW-UP (same question, just remove the “…if ever?”)* | Years ago   1. *Never had exam* |  |

Time End :

Section 5000: Health Care Utilization

Time Begin :

I would now like to know about your recent experiences with obtaining health care from health care workers, hospitals, clinics and the health care system. I want to know if you needed health care recently, and if so, why you needed health care and what type of facility and health care provider you received care from.

| Q5001 | When was the last time that you needed health care?  *INTERVIEWER: this can be inpatient or outpatient care. If less than one month ago, enter “00” for years, "00" for months and enter the number of days.* | years ago months ago*…*  days ago  *98 Never ……………………………. …….🡺*  *-8 DK* | *Q5046* |
| --- | --- | --- | --- |
|  | ***Q5001a.*** *If 'don’t know',*  Was it more than 3 years ago? | 1. *Yes ………………............................…🡺* 2. *No* | *Q5046* |
| Q5002 | The last time you needed health care, did you get health care? | 1. *Yes* 2. *No ………………………………...……🡺* | *Q5025* |

| Q5004 | Thinking about health care you needed in the last 3 years, where did you go most often when you felt sick or needed to consult someone about your health?  *interviewe****r:*** *Only one answer allowed.* | 1. Private doctor’s office 2. Private clinic or health care facility 3. Private hospital 4. Public clinic or health care facility 5. Public hospital 6. Charity or church run clinic 7. Charity or church run hospital 8. Traditional healer [use local term] 9. Pharmacy or dispensary   *87 Other, specify:* |
| --- | --- | --- |

inpatient hospital care

The next two questions ask about any overnight stay in a hospital or other health care facility you have had in the last 3 years.

| Q5005 | In the last 3 years, have you ever stayed overnight in a hospital or long-term care facility? | 1. *Yes, a hospital* 2. *Yes, long term care facility* 3. *Both ( hospital and long term care facility)* 4. *No*  *🡺* | *Q5025* |
| --- | --- | --- | --- |
| Q5006 | When was the last overnight stay in a hospital or long-term care facility?  *INTERVIEWER: If less than one month ago, enter “00” for years, "00" for months and enter number of days.* | years ago months ago days ago  *-8 DK*  *If more than 3 years ago …………………...🡺* | *Q5025* |

Now I would like to know about more recent times - if you've had any overnight stays in a hospital or other type of health care facility in the last 12 months.

| Q5007 | Over the last 12 months, how many different times were you a patient in a hospital/long-term care facility for at least one night? | TIMES  *-8 DK*  *If "00" (no overnight stays) ……….🡺* | *Q5025* |
| --- | --- | --- | --- |

*INPATIENT HOSPITAL CARE Continued…*

I want to know more about why you needed an overnight stay in a health care facility. Starting with the most recent stay, I want to know more about your overnight stays, including why you needed to be hospitalized each time. But first I would like you to come back to thinking about your last overnight hospital stay only.

| Q5008 | What type of hospital or facility was it? Remember we are asking now about your last (most recent) overnight stay.  *INTERVIEWER: One answer only.* | | 1. *Public hospital* 2. *Private hospital* 3. *Charity or church-run hospital* 4. *Old person's home or long-term care facility*   *7 Other, specify:* |  | | |
| --- | --- | --- | --- | --- | --- | --- |
|  | Q5008a. What was the name of this hospital or facility*?* | | *____________________________________________* | | | |
|  | Q5008b. Which reason best describes why you were last hospitalised?  *Interviewer: Respondent can select only ONE main reason for visit. USE SHOWCARD.* | | | | | |
|  | 1. *Communicable disease (infections, malaria, tuberculosis, HIV)* 2. *Maternal and perinatal conditions (pregnancy)* 3. *Nutritional deficiencies* 4. *Acute conditions (diarrhoea, fever, flu, headaches, cough, other)* 5. *Injury (not occupation related)* 6. *Surgery* 7. *Sleep problems* 8. *Occupation/work related condition/injury* 9. *Chronic pain in your joints/arthritis (joints, back, neck)* | 1. *Diabetes or related complications* 2. *Problems with your heart including unexplained pain in chest* 3. *Problems with your mouth, teeth or swallowing* 4. *Problems with your breathing* 5. *High blood pressure / hypertension* 6. *Stroke/sudden paralysis of one side of body* 7. *Generalized pain (stomach, muscle or other nonspecific pain)* 8. *Depression or anxiety* 9. *Cancer*   *87 Other, specify:* | | |  | |
| Q5009 | How did you get there?  *Interviewer: Circle all that the respondent mentions.* | 1. *Private vehicle* 2. *Public transportation* 3. *Taxicab* 4. *Ambulance or emergency vehicle* 5. *Bicycle* 6. *Walked*   *8 DK* | | |  | |
|  | **Q5009a.** About how long did it take you to get there? | : *Hours:Minutes*  *-8 DK* | | | | |
| Q5010 | Who paid for this hospitalisation?  Anyone else?  *interviewer: Circle all responses. Probe to see if anyone else paid or contributed to paying for the care?* | 1. *Respondent* 2. *Spouse/partner* 3. *Son/daughter* 4. *Other family member* 5. *Non-family member* 6. *Mandatory Insurance scheme* 7. *Voluntary Insurance Scheme* 8. *Hospitalisation was free* ***……*** *🡺* | | | | *Q5013* |

*INPATIENT HOSPITAL CARE Continued…*

| Q5011 | Thinking about your last [hospital] stay, how much did you or your family/household members pay out-of-pocket for:  *Interviewer: enter "0" if the service was free - If a person did not have medicines or tests, enter 99998 for “Not applicable, did not have”.*  [use local currency] | a. [Health care provider's] fees |  |
| --- | --- | --- | --- |
|  |  | b. Medicines |  |
|  |  | c. Tests |  |
|  |  | d. Transport |  |
|  |  | e. Other  Specify: |  |
| Q5012 | About how much in total did you or a family/household member pay out-of-pocket for this hospitalisation? |  |  |
| Q5013 | Overall, how satisfied were you with the care you received during your last [hospital] stay? | 1. Very satisfied 2. Satisfied 3. Neither satisfied nor dissatisfied 4. Dissatisfied 5. Very dissatisfied |  |
| Q5014 | What was the outcome or result of your visit to the [hospital]? Did your condition… | 1. Get much better 2. Get better 3. No change 4. Get worse 5. Get much worse |  |
| Q5015 | Was this the outcome or result you had expected? | 1. Yes 2. No |  |

*INTERVIEWER: We will ask the respondent about up to 2 additional overnight stays using Q5016 to Q5017 below. If only ONE overnight stay in the last 12 months, skip to Q5018.*

I have asked you many questions about your last overnight stay, but now I want to know about other overnight stays you have had in the last 12 months. Think now of the overnight stay the time before the one you just described to me. This would be your second overnight stay in the last 12 months.

| Q5016 | What type of hospital or facility was it? | 1. Public hospital 2. Private hospital 3. Charity or church-run hospital 4. Old person's home or long-term care facility   *7 Other, specify:* |  |
| --- | --- | --- | --- |
|  | Q5016a. Which reason best describes why you were last hospitalised?  *Interviewer: Respondent may select only ONE main reason for visit. USE SHOWCARD.* | |  |
|  | 1. *Communicable disease (infections, malaria, tuberculosis, HIV)* 2. *Maternal and perinatal conditions (pregnancy)* 3. *Nutritional deficiencies* 4. *Acute conditions (diarrhoea, fever, flu, headaches, cough, other)* 5. *Injury* 6. *Surgery* 7. *Sleep problems* 8. *Occupation/work related condition/injury* 9. *Chronic pain in your joints/arthritis (joints, back, neck)* | 1. *Diabetes or related complications* 2. *Problems with your heart including unexplained pain in chest* 3. *Problems with your mouth, teeth or swallowing* 4. *Problems with your breathing* 5. *High blood pressure / hypertension* 6. *Stroke/sudden paralysis of one side of body* 7. *Generalized pain (stomach, muscle or other nonspecific pain)* 8. *Depression or anxiety* 9. *Cancer*   *87 Other, specify:* |  |

*INTERVIEWER: if only TWO overnight stays in the last 12 months, skip now to Q5018.*

And now think of the overnight stay the time before the one you just described to me. This would be your third overnight stay in the last 12 months.

| Q5017 | What type of hospital or facility was it? | 1. Public hospital 2. Private hospital 3. Charity or church-run hospital 4. Old person's home or long-term care facility   *7 Other, specify:* |
| --- | --- | --- |
|  | Q5017a. Which reason best describes why you were last hospitalised?  *Interviewer: Respondent can select only ONE main reason for visit. USE SHOWCARD.* | |
|  | 1. *Communicable disease (infections, malaria, tuberculosis, HIV)* 2. *Maternal and perinatal conditions (pregnancy)* 3. *Nutritional deficiencies* 4. *Acute conditions (diarrhoea, fever, flu, headaches, cough, other)* 5. *Injury* 6. *Surgery* 7. *Sleep problems* 8. *Occupation/work related condition/injury* 9. *Chronic pain in your joints/arthritis (joints, back, neck)* | 1. *Diabetes or related complications* 2. *Problems with your heart including unexplained pain in chest* 3. *Problems with your mouth, teeth or swallowing* 4. *Problems with your breathing* 5. *High blood pressure / hypertension* 6. *Stroke/sudden paralysis of one side of body* 7. *Generalized pain (stomach, muscle or other nonspecific pain)* 8. *Depression or anxiety* 9. *Cancer*   *87 Other, specify:* |

*INPATIENT HOSPITAL CARE Continued…*

Now I want you to think again about your most recent overnight stay. I would like to ask you about your impressions of your last overnight stay. I would like you to rate your experiences using the following questions.

| For your last visit to a hospital or long-term care facility, how would you rate the following: | | Very good | Good | Moderate | Bad | Very bad |
| --- | --- | --- | --- | --- | --- | --- |
| Q5018 | ... the amount of time you waited before being attended to? | *1* | *2* | *3* | *4* | *5* |
| Q5019 | ...your experience of being treated respectfully? | *1* | *2* | *3* | *4* | *5* |
| Q5020 | …how clearly health care providers explained things to you? | *1* | *2* | *3* | *4* | *5* |
| Q5021 | …your experience of being involved in making decisions for your treatment? | *1* | *2* | *3* | *4* | *5* |
| Q5022 | ...the way the health services ensured that you could talk privately to providers? | *1* | *2* | *3* | *4* | *5* |
| Q5023 | ...the ease with which you could see a health care provider you were happy with? | *1* | *2* | *3* | *4* | *5* |
| Q5024 | ...the cleanliness in the health facility? | *1* | *2* | *3* | *4* | *5* |

| Q5025 | In the last 12 months, has there been a time when you needed to stay overnight in a health care facility but did not get that care? | 1. *Yes* 2. *No ………………………………...……🡺* | *Q5026* |
| --- | --- | --- | --- |

|  | Q5025a. What was the main reason you needed care, but did not get care?  *Interviewer: Respondent can select ONLY one main reason for visit. USE SHOWCARD (APPENDIX RESPONSE SCALES)* | | |
| --- | --- | --- | --- |
|  | 1. *Communicable disease (infections, malaria, tuberculosis, HIV)* 2. *Maternal and perinatal conditions (pregnancy)* 3. *Nutritional deficiencies* 4. *Acute conditions (diarrhoea, fever, flu, headaches, cough, other)* 5. *Injury (not work related, see 8 below)* 6. *Surgery* 7. *Sleep problems* 8. *Occupation/work related condition/injury* 9. *Chronic pain in your joints/arthritis (joints, back, neck)* 10. *Diabetes or related complications* | 1. *Problems with your heart including unexplained pain in chest* 2. *Problems with your mouth, teeth or swallowing* 3. *Problems with your breathing* 4. *High blood pressure / hypertension* 5. *Stroke/sudden paralysis of one side of body* 6. *Generalized pain (stomach, muscle or other nonspecific pain)* 7. *Depression or anxiety* 8. *Cancer*   *87 Other, specify:* |  |
|  | **Q5025b.** Which reason(s) best explains why you did not get health care?  *Interviewer:*  *Circle all that the respondent indicates.* | 1. Could not afford the cost of the visit 2. No transport available 3. Could not afford the cost of transport 4. You were previously badly treated 5. Could not take time off work or had other commitments 6. The health care provider's drugs or equipment were inadequate 7. The health care provider's skills were inadequate 8. You did not know where to go 9. You tried but were denied health care 10. You thought you were not sick enough   *87 Other, specify:* |  |

Outpatient care and Care at Home

Now I will shift away from questions about overnight stays – to questions about health care you received that did not include an overnight hospital stay. The following questions are about care you received at a hospital, health centre, clinic, private office or at home from a health care provider, but where you did not stay overnight.

| Q5026 | Over the last 12 months, did you receive any health care NOT including an overnight stay in hospital or long-term care facility? | 1. *Yes* 2. *No*  *🡺* | *Q5046* |
| --- | --- | --- | --- |
| Q5027 | In total, how many times did you receive health care or consultation in the last 12 months? | Times |  |

Now I would like you to think about the most recent visit - and ask you specifically about your last or most recent visit.

| Q5028 | What was the last (most recent) health care facility you visited in the last 12 months?  ***interviewer:***  *Read out responses, circle one option only* | 1. Private doctor’s office 2. Private clinic or health care facility 3. Private hospital 4. Public clinic or health care facility 5. Public hospital 6. Charity or church run clinic 7. Charity or church run hospital 8. Home visit   *87 Other, specify:* |
| --- | --- | --- |
|  | Q5028a. What was the name of this health care facility or provider? | *____________________________________________* |

*Outpatient care and Care at Home continued…*

| Q5029 | Which was the last (most recent) health care provider you visited?  *Interviewer:*  *After Q5029 substitute the type of health care provider selected by the patient when you see [health care provider] in parentheses* | 1. Medical doctor (including surgeon, gynecologist, psychiatrist, ophthalmologist,…) 2. Nurse/Midwife 3. Dentist 4. Physiotherapist or chiropractor 5. Traditional medicine practitioner (use local name) 6. Pharmacist, druggist 7. Home health care worker   *8 DK* | |
| --- | --- | --- | --- |
|  | Q5029a. What was the sex of the [health care provider]? | 1. *Male* 2. *Female* | |
|  | Q5029b. Was this visit to [health care provider] for a chronic (ongoing) condition, new condition, both or routine check-up? | 1. Chronic 2. New 3. Both 4. Routine check-up | |
|  | Q5029c. Which reason best describes why you needed this visit?  *Interviewer: Respondent can select only ONE main reason for visit. USE SHOWCARD.* | |  |
|  | 1. *Communicable disease (infections, malaria, tuberculosis, HIV)* 2. *Maternal and perinatal conditions (pregnancy)* 3. *Nutritional deficiencies* 4. *Acute conditions (diarrhoea, fever, flu, headaches, cough, other)* 5. *Injury* 6. *Surgery* 7. *Sleep problems* 8. *Occupation/work related condition/injury* 9. *Chronic pain in your joints/arthritis (joints, back, neck)* | 1. *Diabetes or related complications* 2. *Problems with your heart including unexplained pain in chest* 3. *Problems with your mouth, teeth or swallowing* 4. *Problems with your breathing* 5. *High blood pressure / hypertension* 6. *Stroke/sudden paralysis of one side of body* 7. *Generalized pain (stomach, muscle or other nonspecific pain)* 8. *Depression or anxiety* 9. *Cancer*   *87 Other, specify:* | |
| Q5030 | Thinking about your last visit, how did you get there?  *Interviewer:*  *Circle all that the respondent mentions.* | 1. *Private vehicle* 2. *Public transportation* 3. *Taxicab* 4. *Ambulance or emergency vehicle* 5. *Bicycle* 6. *Walked*   *8 DK*  *9 Not applicable* | |
| Q5031 | About how long did it take you to get there? | : *Hours:Minutes*  *-8 DK* | |
| Q5032 | Who paid for this most recent visit?  Anyone else?  *interviewer: Circle all responses. Probe to see if anyone else paid or contributed to paying for the care?* | 1. *Respondent* 2. *Spouse/partner* 3. *Son/daughter* 4. *Other family member* 5. *Non-family member* 6. *Mandatory Insurance Scheme* 7. *Voluntary Insurance Scheme* 8. *It was free* *…… 🡺* | *Q5034* |

*Outpatient care and Care at Home continued…*

| Q5033 | Thinking about your last visit, how much did you or your household pay for:  *Interviewer: Only write "0" if the service was free. If a person did not have tests or drugs, enter 99998 for “Not applicable, did not have”.*  *(local currency)* | a. [Health care provider's] fees |
| --- | --- | --- |
|  |  | b. Medicines |
|  |  | c. Tests |
|  |  | d. Transport |
|  |  | e. Other , specify: |
|  |  | F. Total costs |
| Q5034 | Overall, how satisfied were you with the care you received during your last visit? | 1. Very satisfied 2. Satisfied 3. Neither satisfied nor dissatisfied 4. Dissatisfied 5. Very dissatisfied |
| Q5035 | What was the outcome of your visit to the health care provider? Did your condition….? | 1. Get much better 2. Get better 3. No change 4. Get worse 5. Get much worse |
| Q5036 | Was this the outcome/result you had expected? | 1. *Yes* 2. *No* |

*INTERVIEWER: We will ask the respondent about up to two additional visits using Q5037 through Q5038. If only ONE visit in past 12 months, skip to Q5039.*

I will ask you about up to two more encounters/visits with health professionals in addition to the last visit you just described. So could you please tell us now about the visit prior to the last (most recent) visit you just described. This would describe your second to last visit.

| Q5037 | Which was the health care provider you visited?  Interviewer:  *After Q5037 substitute the type of health care provider selected by the patient when you see [health care provider] in parentheses* | 1. Medical doctor (including surgeon, gynecologist, psychiatrist, ophthalmologist, etc.) 2. Nurse/Midwife 3. Dentist 4. Physiotherapist or chiropractor 5. Traditional medicine practitioner (*use local name*) 6. Pharmacist, druggist 7. Home health care worker   8 *DK* |
| --- | --- | --- |

*Outpatient care and Care at Home continued…*

|  | **Q5037a**. What was the sex of the [*health care provider*]? | 1. Male 2. Female |
| --- | --- | --- |
|  | **Q5037b.** Was this visit to [*health care provider*] for a chronic (ongoing) condition, new condition, both or routine check-up? | 1. Chronic 2. New 3. Both 4. Routine check-up |
|  | **Q5037c.** Which reason best describes why you needed this visit?  *Interviewer: Respondent may select only ONE main reason for visit. USE SHOWCARD* | |
|  | 1. Communicable disease (infections, malaria, tuberculosis, HIV) 2. Maternal and perinatal conditions (pregnancy) 3. Nutritional deficiencies 4. Acute conditions (diarrhoea, fever, flu, headaches, cough, other) 5. Injury 6. Surgery 7. Sleep problems 8. Occupation/work related condition/injury 9. Chronic pain in your joints/arthritis (joints, back, neck) | 1. Diabetes or related complications 2. Problems with your heart including unexplained pain in chest 3. Problems with your mouth, teeth or swallowing 4. Problems with your breathing 5. High blood pressure / hypertension 6. Stroke/sudden paralysis of one side of body 7. Generalized pain (stomach, muscle or other nonspecific pain) 8. Depression or anxiety 9. Cancer   87 Other, specify: |

*INTERVIEWER: if just TWO visits in last 12 months, skip to Q5039.*

And now think of the visit the time before the one you just described to me. This would be your third visit in the last 12 months.

| Q5038 | Which was the health care provider you visited?  *Interviewer:*  *After Q5038 substitute the type of health care provider selected by the patient when you see [health care provider] in parentheses* | 1. Medical doctor (including surgeon, gynecologist, psychiatrist, ophthalmologist, etc.) 2. Nurse/Midwife 3. Dentist 4. Physiotherapist or chiropractor 5. Traditional medicine practitioner *(use local name)* 6. Pharmacist, druggist 7. Home health care worker   8 *DK* |
| --- | --- | --- |
|  | **Q5038a**. What was the sex of the [*health care provider*]? | 1. Male 2. Female |
|  | **Q5038b**. Was this visit to [*health care provider*] for a chronic (ongoing) condition, new condition, both or routine check-up? | 1. Chronic 2. New 3. Both 4. Routine check-up |

*Outpatient care and Care at Home continued…*

|  | **Q5038c**. Which reason best describes why you needed this visit?  *Interviewer: Respondent can select only one main reason for visit. USE SHOWCARD.* | |
| --- | --- | --- |
|  | 1. Communicable disease (infections, malaria, tuberculosis, HIV) 2. Maternal and perinatal conditions (pregnancy) 3. Nutritional deficiencies 4. Acute conditions (diarrhoea, fever, flu, headaches, cough, other) 5. Injury 6. Surgery 7. Sleep problems 8. Occupation/work related condition/injury 9. Chronic pain in your joints/arthritis (joints, back, neck) | 1. Diabetes or related complications 2. Problems with your heart including unexplained pain in chest 3. Problems with your mouth, teeth or swallowing 4. Problems with your breathing 5. High blood pressure / hypertension 6. Stroke/sudden paralysis of one side of body 7. Generalized pain (stomach, muscle or other nonspecific pain) 8. Depression or anxiety 9. Cancer   87 Other, specify: |

Now I would like you to think about your most recent visit again. I want to know your impressions of your most recent visit for health care. I would like you to rate your experiences using the following questions.

| For your last visit to a health care provider, how would you rate the following: | | *Very good* | *Good* | *Moderate* | *Bad* | *Very bad* |
| --- | --- | --- | --- | --- | --- | --- |
| Q5039 | ... the amount of time you waited before being attended to? | *1* | *2* | *3* | *4* | *5* |
| Q5040 | ...your experience of being treated respectfully? | *1* | *2* | *3* | *4* | *5* |
| Q5041 | …how clearly health care providers explained things to you? | *1* | *2* | *3* | *4* | *5* |
| Q5042 | …your experience of being involved in making decisions for your treatment? | *1* | *2* | *3* | *4* | *5* |
| Q5043 | ...the way the health services ensured that you could talk privately to providers? | *1* | *2* | *3* | *4* | *5* |
| Q5044 | ...the ease with which you could see a health care provider you were happy with? | *1* | *2* | *3* | *4* | *5* |
| Q5045 | ...the cleanliness in the health facility? | *1* | *2* | *3* | *4* | *5* |

| Q5046 | In the last 12 months was there a time when you needed health care from a doctor/in a clinic, but did not get care? | 1. *Yes* 2. *No ………………………………...……🡺* | *Q5053* |
| --- | --- | --- | --- |

|  | Q5046a. What was the main reason you needed care, even if you did not get care?  *Interviewer: Respondent can select ONLY one main reason for visit. USE SHOWCARD (APPENDIX RESPONSE SCALES)* | | |
| --- | --- | --- | --- |
|  | 1. *Communicable disease (infections, malaria, tuberculosis, HIV)* 2. *Maternal and perinatal conditions (pregnancy)* 3. *Nutritional deficiencies* 4. *Acute conditions (diarrhoea, fever, flu, headaches, cough, other)* 5. *Injury (not work related, see 8 below)* 6. *Surgery* 7. *Sleep problems* 8. *Occupation/work related condition/injury* 9. *Chronic pain in your joints/arthritis (joints, back, neck)* 10. *Diabetes or related complications* | 1. *Problems with your heart including unexplained pain in chest* 2. *Problems with your mouth, teeth or swallowing* 3. *Problems with your breathing* 4. *High blood pressure / hypertension* 5. *Stroke/sudden paralysis of one side of body* 6. *Generalized pain (stomach, muscle or other nonspecific pain)* 7. *Depression or anxiety* 8. *Cancer*   *87 Other, specify:* |  |
|  | **Q5046b.** Which reason(s) best explains why you did not get health care?  *Interviewer:*  *Circle all that the respondent indicates.* | 1. Could not afford the cost of the visit 2. No transport available 3. Could not afford the cost of transport 4. You were previously badly treated 5. Could not take time off work or had other commitments 6. The health care provider's drugs or equipment were inadequate 7. The health care provider's skills were inadequate 8. You did not know where to go 9. You tried but were denied health care 10. You thought you were not sick enough   *87 Other, specify:* |  |

We would like to finish this section by asking you two questions about your satisfaction with the health system in your country. If you received health care, think about the health care service(s) you received in the last 12 months when answering the questions.]

| Q5053 | In general, how satisfied are you with how the health care services are run in your country [in your area] – are you very satisfied, satisfied, neither satisfied nor dissatisfied, fairly dissatisfied, or very dissatisfied? | 1. Very satisfied 2. Satisfied 3. Neither satisfied nor dissatisfied 4. Dissatisfied 5. Very Dissatisfied |
| --- | --- | --- |
| *Q5054* | How would you rate the way health care in your country involves you in deciding what services it provides and where it provides them? | 1. Very good 2. Good 3. Moderate 4. Bad 5. Very bad |

Time End :

Section 6000: Social Networks

Time Begin :

We would like to shift away from questions about your direct health. This section of the survey asks your opinions about other areas and issues in your life. The following questions are to get your opinions about community, social and political aspects in your life.

We’d like to know about some of your involvement in your community. For all of these, I want you just to give me your best guess, and don’t worry that you might be off a little.

|  | *How often in the last 12 months have you …* | *Never* | *Once or twice per year* | *Once or twice per month* | *Once or twice per week* | *Daily* |
| --- | --- | --- | --- | --- | --- | --- |
| Q6001 | … attended any public meeting in which there was discussion of local or school affairs? | *1* | *2* | *3* | *4* | *5* |
| Q6002 | … met personally with someone you consider to be a community leader? | *1* | *2* | *3* | *4* | *5* |
| Q6003 | …attended any group, club, society, union or organizational meeting? | *1* | *2* | *3* | *4* | *5* |
| Q6004 | … worked with other people in your neighborhood to fix or improve something? | *1* | *2* | *3* | *4* | *5* |
| Q6005 | … had friends over to your home? | *1* | *2* | *3* | *4* | *5* |
| Q6006 | … been in the home of someone who lives in a different neighbourhood than you do or had them in your home? | *1* | *2* | *3* | *4* | *5* |
| Q6007 | … socialized with coworkers outside of work? | *1* | *2* | *3* | *4* | *5* |
| Q6008 | … attended religious services (not including weddings and funerals)? | *1* | *2* | *3* | *4* | *5* |
| Q6009 | … gotten out of the house/your dwelling to attend social meetings, activities, programs or events or to visit friends or relatives? | *1* | *2* | *3* | *4* | *5* |
| Q6010 | …communicated with your closest friends? | *1* | *2* | *3* | *4* | *5* |

The next questions are about how you feel about different aspects of your life. For each one, tell me how often you feel that way.

|  | | Never | Rarely | Sometimes | Often |
| --- | --- | --- | --- | --- | --- |
| Q6011a | First, how often do you feel that you lack companionship? | 1 | 2 | 3 | 4 |
| Q6011b | How often do you feel left out? | 1 | 2 | 3 | 4 |
| Q6011c | How often do you feel isolated from others? | 1 | 2 | 3 | 4 |

We’d like to ask you a few questions about how you view other people and institutions.

| Q6012 | Generally speaking, would you say that most people can be trusted or that you can't be too careful in dealing with people? | 1. Can be trusted 2. Can't be too careful |
| --- | --- | --- |
| Q6013 | Do you have someone you can trust and confide in? | 1. Yes 2. No |

Next, we'd like to know how much you trust different groups of people.

|  | | To a very great extent | To a great extent | Neither great nor small extent | To a small extent | To a very small extent |
| --- | --- | --- | --- | --- | --- | --- |
| Q6014 | First, think about people in your neighbourhood. Generally speaking, would you say that you can trust them…? | 1 | 2 | 3 | 4 | 5 |
| Q6015 | Now, think about people whom you work with. Generally speaking, would you say that you can trust them …? | 1 | 2 | 3 | 4 | 5 |
| Q6016 | And how about strangers? Generally speaking, would you say that you can trust them …? | 1 | 2 | 3 | 4 | 5 |

For the last three questions in this section, we ask about safety in the area where you live.

| Q6017 | In general, how safe from crime and violence do you feel when you are alone at home? | 1. Completely safe 2. Very safe 3. Moderately safe 4. Slightly safe 5. Not safe at all |
| --- | --- | --- |
| Q6018 | How safe do you feel when walking down your street alone after dark? | 1. Completely safe 2. Very safe 3. Moderately safe 4. Slightly safe 5. Not safe at all |
| Q6019 | In the last 12 months, have you or anyone in your household been the victim of a violent crime, such as assault or mugging? | 1. Yes 2. No |

Time End :

Section 7000: Subjective Well-Being and Quality of Life

Time Begin :

| Now, we'd like to ask for your thoughts about your life and life situation. We want to know how you feel about your health and quality of life. | | | | | | | |
| --- | --- | --- | --- | --- | --- | --- | --- |
| Q7001 | Do you have enough energy for everyday life? | | | 1. Completely 2. Mostly 3. Moderately 4. A little 5. None at all | | | |
| Q7002 | Do you have enough money to meet your needs? | | | 1. Completely 2. Mostly 3. Moderately 4. A little 5. None at all | | | |
| *Please tell us how satisfied you are with the following issues.* | | | | | | | |
|  | How satisfied are you with… | *Very satisfied* | *Satisfied* | | *Neither satisfied nor dissatisfied* | *Dissatisfied* | *Very Dissatisfied* |
| Q7003 | … your health? | *1* | *2* | | *3* | *4* | *5* |
| Q7004 | … yourself? | *1* | *2* | | *3* | *4* | *5* |
| Q7005 | … your ability to perform your daily living activities? | *1* | *2* | | *3* | *4* | *5* |
| Q7006 | … your personal relationships? | *1* | *2* | | *3* | *4* | *5* |
| Q7007 | … the conditions of your living place? | *1* | *2* | | *3* | *4* | *5* |
| Q7008 | Taking all things together, how satisfied are you with your life as a whole these days? | *1* | *2* | | *3* | *4* | *5* |

| Q7008a | How often have you felt that you were unable to control the important things in your life?  *Read responses* | 1. Never 2. Almost never 3. Sometimes 4. Fairly often 5. Very often |
| --- | --- | --- |
| Q7008b | How often have you found that you could not cope with all the things that you had to do?  *Read responses* | 1. Never 2. Almost never 3. Sometimes 4. Fairly often 5. Very often |
| Q7009 | How would you rate your overall quality of life?  *Read responses* | 1. Very Good 2. Good 3. Moderate 4. Bad 5. Very Bad   8 *DK* |

| Q7010 | Taking all things together, how would you say you are these days?  *Read responses* | 1. Very happy 2. Happy 3. Neither happy nor unhappy 4. Unhappy 5. Very unhappy   *8 DK* |
| --- | --- | --- |
| Q7011 | Please imagine a ladder with steps numbered from one at the bottom to 10 at the top.  The top of the ladder represents the best possible life for you and the bottom of the ladder represents the worst possible life for you.  On which step of the ladder would you say you personally feel you stand at this time?  *Interviewer: Show respondent the ladder* | NUMBER________  DK 88  REFUSED 97 |
| Q7012 | On which step do you think you will stand about five (5) years from now?  *Interviewer: Show respondent the ladder* | NUMBER________  DK 88  REFUSED 97 |


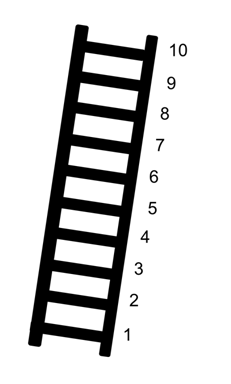


10 = The best possible life for you

1 = The worst possible life for you

Day reconstruction – Summary Full Day

| *INTERVIEWER: For this module, you will ask the respondent to reconstruct his or her entire previous day beginning from when s/he woke up until s/he went to sleep. You will not record the day in an event-by-event manner. You will only record broadly what was done in the morning, afternoon and evening. You will also ask the respondent how s/he felt during these 3 parts of the day.*  *In addition, you will ask details about one experience from each part of the day.* |
| --- |

| INTRODUCTION to Day Reconstruction - Full Day | | |
| --- | --- | --- |
| Now I would like to ask you questions about what you did yesterday. I want you to try to remember the sequence of activities that you did from when you woke up until when you went to sleep last night.  I will start by asking you what you did in the morning yesterday, and you should just give me a short description. Then I will ask about the afternoon and then the evening. | | |
| Q7013 | At what time did you wake up yesterday?  *INTERVIEWER: If respondent can’t remember, get his or her best guess.* | : TIME |
| Q7014 | At what time did you go to sleep yesterday?  *INTERVIEWER: If respondent can’t remember, get his or her best guess.* | : TIME |

| *INTERVIEWER: Please note for Q7015 and Q7016, Q7050 and Q7051, Q7100 and Q7101 :*   - *Circle all activities that the person spontaneously mentions.* - *You do not need to record the order and you do not need to record an item that is repeated.* - *This does not have to be comprehensive. It is just meant to be an approximation.* - *Please also circle all people that they say they were with.* - *If the person takes more than 3 minutes to tell you about their morning/afternoon/evening experiences, you should ask them to give you less detail.* |
| --- |

Morning

| Q7015 | Please tell me the main things that you did yesterday morning from the time you woke up until around noon/mid-day. Please also mention if you were talking or interacting with anyone for any parts of the morning. By interacting with, I mean were you consistently paying attention to someone. For example, if you were bathing a young child you would be interacting with them even if you were not talking. On the other hand, talking to someone for less than 5 minutes does not count as interacting.  Be sure to cover as much as you can remember. You don’t have to go in order, but it’s probably easier that way. Be sure to stop with activities from around noon/mid-day. | | |
| --- | --- | --- | --- |
| 1 Working  2 Preparing food  3 Doing housework  4 Subsistence farming  5 Watching children  6 Shopping  7 Walking somewhere  8 Traveling by bicycle  9 traveling by car/bus/train | | 10 Rest (includes tea/coffee break)  11 Chatting with Someone  12 Playing (includes cards/games)  13 Reading  14 Listening to radio  15 Watching TV  16 Exercising or Leisurely Walk  17 Other Leisurely Activity | 18 Grooming or Bathing  19 Eating  20 Religious activity  21 Providing care to someone  22 Intimate Relations/Sex  23 Went to sleep for the night  24 Medical consultation |

CIRCLE ALL responses in Q7015

|  |  |  |
| --- | --- | --- |
|  |  |  |
|  |  |  |
|  |  |  |

| Q7025 | Random activity selected from the morning. |  |
| --- | --- | --- |
| Q7026 | Now I want you to think about the XXX (from Q7015) you mentioned during the morning.  How long did this activity last? | : Hours : Minutes |
| Q7027 | At what time did this activity begin?  *INTERVIEWER: If respondent has trouble with exact time, get estimate or approximate.* | : TIME |
| Q7028 | Were you talking or interacting with anyone when you did this? By interacting with, I mean were you consistently paying attention to someone. For example, if you were bathing a young child you would be interacting with them even if you were not talking. On the other hand, talking to someone for less than 5 minutes does not count as interacting.  *INTERVIEWER: Respondent may provide more than one answer - circle responses.* | 1. Alone ……..……🡺 Q7029 2. Spouse 3. Adult Children 4. Young Children or Grandchildren 5. Family (Other than Spouse/Children) 6. Friends 7. Co-Workers   87 Other, specify: |
|  | Q7028a. At the time, how friendly were you feeling towards this person (these people)? | 1. Very friendly 2. A little friendly 3. A little irritated 4. Very irritated |

| Please think about how you felt yesterday morning during your XXX (Q7015). Rate your feelings from 0 to 6 where 0 means you did not feel like that at all and 6 means you felt very much like that. | | | | | | | | |
| --- | --- | --- | --- | --- | --- | --- | --- | --- |
|  |  | Not at all |  |  |  |  |  | Very much |
| Q7029 | How worried were you feeling? | 0 | 1 | 2 | 3 | 4 | 5 | 6 |
| Q7030 | How rushed were you feeling? | 0 | 1 | 2 | 3 | 4 | 5 | 6 |
| Q7031 | How irritated or angry were you feeling? | 0 | 1 | 2 | 3 | 4 | 5 | 6 |
| Q7032 | How depressed were you feeling? | 0 | 1 | 2 | 3 | 4 | 5 | 6 |
| Q7033 | How tense or stressed were you feeling? | 0 | 1 | 2 | 3 | 4 | 5 | 6 |
| Q7034 | How calm or relaxed were you feeling? | 0 | 1 | 2 | 3 | 4 | 5 | 6 |
| Q7035 | How much were you enjoying what you were doing? | 0 | 1 | 2 | 3 | 4 | 5 | 6 |

Afternoon

| Q7050 | Please tell me the main things that you did yesterday afternoon from around noon/mid-day until evening time (around 18.00 or 6pm). Please also mention if anyone was with you for any parts of the afternoon.  Be sure to cover as much as you can remember. You don’t have to go in order, but it’s probably easier that way. Be sure to describe only the activities from your afternoon yesterday between mid-day and evening. | | |
| --- | --- | --- | --- |
| 1 Working  2 Preparing food  3 Doing housework  4 Subsistence farming  5 Watching children  6 Shopping  7 Walking somewhere  8 Traveling by bicycle  9 traveling by car/bus/train | | 10 Rest (includes tea/coffee break)  11 Chatting with Someone  12 Playing (includes cards/games)  13 Reading  14 Listening to radio  15 Watching TV  16 Exercising or Leisurely Walk  17 Other Leisurely Activity | 18 Grooming or Bathing  19 Eating  20 Religious activity  21 Providing care to someone  22 Intimate Relations/Sex  23 Went to sleep for the night  24 Medical consultation |

CIRCLE ALL responses in Q7050

|  |  |  |
| --- | --- | --- |
|  |  |  |
|  |  |  |
|  |  |  |

| Q7065 | Random activity selected from the afternoon. |  |
| --- | --- | --- |
| Q7066 | Now I want you to think about the YYY (from Q7050) you mentioned during the afternoon.  How long did this activity last? | : Hours : Minutes |
| Q7067 | At what time did this activity begin?  *INTERVIEWER: If respondent has trouble with exact time, get estimate or approximate.* | : TIME |
| Q7068 | Were you talking or interacting with anyone when you did this? By interacting with, I mean were you consistently paying attention to someone. For example, if you were bathing a young child you would be interacting with them even if you were not talking. On the other hand, talking to someone for less than 5 minutes does not count as interacting.  *INTERVIEWER: Respondent may provide more than one answer - circle responses.* | 1. Alone ……..……🡺 Q7069 2. Spouse 3. Adult Children 4. Young Children or Grandchildren 5. Family (Other than Spouse/Children) 6. Friends 7. Co-Workers   87 Other, specify: |
|  | Q7068a. At the time, how friendly were you feeling towards this person (these people)? | 1. Very friendly 2. A little friendly 3. A little irritated 4. Very irritated |

| Please think about how you felt yesterday afternoon during your YYY (Q7050. Rate your feelings from 0 to 6 where 0 means you did not feel like that at all and 6 means you felt very much like that. | | | | | | | | |
| --- | --- | --- | --- | --- | --- | --- | --- | --- |
|  |  | Not at all |  |  |  |  |  | Very much |
| Q7069 | How worried were you feeling? | 0 | 1 | 2 | 3 | 4 | 5 | 6 |
| Q7070 | How rushed were you feeling? | 0 | 1 | 2 | 3 | 4 | 5 | 6 |
| Q7071 | How irritated or angry were you feeling? | 0 | 1 | 2 | 3 | 4 | 5 | 6 |
| Q7072 | How depressed were you feeling? | 0 | 1 | 2 | 3 | 4 | 5 | 6 |
| Q7073 | How tense or stressed were you feeling? | 0 | 1 | 2 | 3 | 4 | 5 | 6 |
| Q7074 | How calm or relaxed were you feeling? | 0 | 1 | 2 | 3 | 4 | 5 | 6 |
| Q7075 | How much were you enjoying what you were doing? | 0 | 1 | 2 | 3 | 4 | 5 | 6 |

Evening

| Q7100 | Please tell me the main things that you did yesterday evening from around 6pm (18.00) until you went to sleep. Please also mention if anyone was with you for any parts of the evening.  Be sure to cover as much as you can remember. You don’t have to go in order, but it’s probably easier that way. | | |
| --- | --- | --- | --- |
| 1 Working  2 Preparing food  3 Doing housework  4 Subsistence farming  5 Watching children  6 Shopping  7 Walking somewhere  8 Traveling by bicycle  9 traveling by car/bus/train | | 10 Rest (includes tea/coffee break)  11 Chatting with Someone  12 Playing (includes cards/games)  13 Reading  14 Listening to radio  15 Watching TV  16 Exercising or Leisurely Walk  17 Other Leisurely Activity | 18 Grooming or Bathing  19 Eating  20 Religious activity  21 Providing care to someone  22 Intimate Relations/Sex  23 Went to sleep for the night  24 Medical consultation |

CIRCLE ALL responses in Q7100

|  |  |  |
| --- | --- | --- |
|  |  |  |
|  |  |  |
|  |  |  |

| Q7110 | Random activity selected from the evening. |  |
| --- | --- | --- |
| Q7111 | Now I want you to think about the ZZZ (from Q7100) you mentioned from yesterday evening.  How long did this activity last? | : Hours : Minutes |
| Q7112 | At what time did this activity begin?  *INTERVIEWER: If respondent has trouble with exact time, get estimate or approximate.* | : TIME |
| Q7113 | Were you talking or interacting with anyone when you did this? By interacting with, I mean were you consistently paying attention to someone. For example, if you were bathing a young child you would be interacting with them even if you were not talking. On the other hand, talking to someone for less than 5 minutes does not count as interacting.  *INTERVIEWER: Respondent may provide more than one answer - circle responses.* | 1. Alone ……..……🡺 Q7114 2. Spouse 3. Adult Children 4. Young Children or Grandchildren 5. Family (Other than Spouse/Children) 6. Friends 7. Co-Workers   87 Other, specify: |
|  | Q7113a. At the time, how friendly were you feeling towards this person (these people)? | 1. Very friendly 2. A little friendly 3. A little irritated 4. Very irritated |

| Please think about how you felt yesterday evening during your ZZZ (Q7100). Rate how you were feeling from 0 to 6 where 0 means you did not feel like that at all and 6 means you felt very much like that. | | | | | | | | |
| --- | --- | --- | --- | --- | --- | --- | --- | --- |
|  |  | Not at all |  |  |  |  |  | Very much |
| Q7114 | How worried were you feeling? | 0 | 1 | 2 | 3 | 4 | 5 | 6 |
| Q7115 | How rushed were you feeling? | 0 | 1 | 2 | 3 | 4 | 5 | 6 |
| Q7116 | How irritated or angry were you feeling? | 0 | 1 | 2 | 3 | 4 | 5 | 6 |
| Q7116 | How depressed were you feeling? | 0 | 1 | 2 | 3 | 4 | 5 | 6 |
| Q7117 | How tense or stressed were you feeling? | 0 | 1 | 2 | 3 | 4 | 5 | 6 |
| Q7118 | How calm or relaxed were you feeling? | 0 | 1 | 2 | 3 | 4 | 5 | 6 |
| Q7119 | How much were you enjoying what you were doing? | 0 | 1 | 2 | 3 | 4 | 5 | 6 |

| I will now ask you some questions about how you felt yesterday overall.  Looking at the whole day (morning, afternoon, AND evening), please tell me whether you had these feelings for much of the day. Please just answer “yes” or “no”. | | |
| --- | --- | --- |
| Q7501 | Did you feel …worried… for much of the day yesterday? Yes or no. | 1. Yes 2. No |
| Q7502 | Did you feel …rushed… for much of the day yesterday? Yes or no. | 1. Yes 2. No |
| Q7503 | Did you feel …irritated or angry…for much of the day yesterday? | 1. Yes 2. No |
| Q7504 | Did you feel …depressed…? | 1. Yes 2. No |
| Q7505 | Did you feel …tense or stressed…? | 1. Yes 2. No |
| Q7506 | Did you feel …calm or relaxed…? | 1. Yes 2. No |
| Q7507 | Were you enjoying what you were doing for much of the day yesterday? | 1. Yes 2. No |
| Q7508 | Did you feel …lonely … for much of the day yesterday? | 1. Yes 2. No |
| Q7509 | Did you feel … bored …? | 1. Yes 2. No |
| Q7510 | Did you feel …physical pain… for much of the day yesterday? | 1. Yes 2. No |
| Q7511 | Did you feel …tired…? | 1. Yes 2. No |
| Q7512 | Did you have a stomach ache at any time yesterday? | 1. Yes 2. No |
| Q7513 | Did you have a headache at any time yesterday? | 1. Yes 2. No |
| Q7514 | Did you smile or laugh a lot yesterday? | 1. Yes 2. No |
| Q7515 | What part of the day did you enjoy most yesterday? Was it the morning, the afternoon, or the evening? | 1. Morning 2. Afternoon 3. Evening |
| Q7516 | Compared to a typical day, how much free time did you have yesterday? Was yesterday typical, or did you have more free time yesterday, or did you have less free time yesterday? | 1. More Free Time 2. Typical 3. Less Free Time |
| Q7517 | Compared to a typical day, how was your mood yesterday? Was it typical, or were you in a better mood yesterday, or were you in a worse mood yesterday? | 1. Better Mood 2. Typical 3. Worse Mood |
| Q7518 | How many hours did you sleep last night? | :Hours : Minutes  -8 *DON'T REMEMBER* |
| Q7519 | Please rate the quality of your sleep last night. Was it very good, good, moderate, poor or very poor? | 1 Very good  2 Good  3 Moderate  4 Poor  5 Very poor |
| Q7520 | How many hours did you sleep the night before last? | :Hours : Minutes  8 *DON'T REMEMBER* |
| Q7521 | Please rate the quality of your sleep the night before last. Was it very good, good, moderate, poor or very poor? | 1 Very good  2 Good  3 Moderate  4 Poor  5 Very poor |
| Q7522 | Who do you think are happier, men or women? Or are they equally happy? | 1. Men 2. Women 3. Equally Happy |
| For the following questions, I will ask you to compare yourself to other people your age who live in this area. | | |
| Q7524 | Compared to other people, are you usually in a better mood or a worse mood or are you about the same? | 1. Better Mood 2. Same Mood 3. Worse Mood |
| Q7526 | Are you more anxious or less anxious than most others? Or are you about the same? | 1 More Anxious  2 Same level  3 Less Anxious |
| Q7527 | Are you more healthy or less healthy than most people your age? Or are you about the same? | 1 More Healthy  2 Same level of health  3 Less Healthy |

Time End :

*INTERVIEWER: This is the end of the interview. Complete section 9000 when you have finished with the respondent.* This completes the interview. We thank you for your time and answers. I have your contact details and may be in touch again. Should you have any questions or concerns please do not hesitate to contact my supervisor [*give supervisor's name*]. As mentioned in the consent form, we may return to you in two years time and would appreciate speaking with you again.

Section 9000: Interviewer Assessment

| *INTERVIEWER* | | | |
| --- | --- | --- | --- |
| *Q9001* | *Was someone else present during the interview?* | *1 Yes* | *2 No* |
|  | *Did respondent have…* | | |
| *Q9002* | *Hearing problem?* | *1 Yes* | *2 No* |
| *Q9003* | *Vision problem?* | *1 Yes* | *2 No* |
| *Q9004* | *Use wheelchair?* | *1 Yes* | *2 No* |
| *Q9005* | *Use cane/crutches/walker?* | *1 Yes* | *2 No* |
| *Q9006* | *Have difficulties walking?* | *1 Yes* | *2 No* |
| *Q9007* | *Paralysis?* | *1 Yes* | *2 No* |
| *Q9008* | *Cough continually?* | *1 Yes* | *2 No* |
| *Q9009* | *Shortness of breath?* | *1 Yes* | *2 No* |
| *Q9010* | *Mental problems?* | *1 Yes* | *2 No* |
| *Q9011* | *Other health problem?* | *1 Yes* | *2 No* |
| *Q9012* | *Amputated limb (arm or leg)?* | *1 Yes* | *2 No* |

| Q9013 | *What is your assessment of the respondent's cooperation?* | 1 Very good  2 Good  3 Moderate  4 Bad  5 Very bad |
| --- | --- | --- |
| Q9014 | *What is your evaluation of the accuracy and completeness of the respondent's answers?* | 1. Very high 2. High 3. Average 4. Low 5. Very low |

|  |  | QUESTION NUMBER(s) | NOTES |
| --- | --- | --- | --- |
| Q9015 | Questions with doubtful answers |  |  |
| Q9016 | Questions needing follow-up or clarification from supervisor |  |  |
| Q9017 | Other problems or issues |  |  |
| Q9018 | What questions did respondent find difficult, embarrassing or confusing? |  |  |
| Q9019 | What questions did you the interviewer find difficult, embarrassing or confusing? |  |  |
| Notes: | | | |
