# Supplementary material for: Disparities in outpatient and inpatient utilization by rural-urban areas among older Mongolians based on a modified WHO-SAGE instrument
Source: BMC Health Serv Res. 2021 Oct 30;21:1183. doi: 10.1186/s12913-021-07156-y (PMC8556801; doi:10.1186/s12913-021-07156-y)
Supplement: Supplementary file 2 — Additional file 2. [file 12913_2021_7156_MOESM2_ESM.docx]

**Supplement Table1 Disparities in outpatient and inpatient utilization stratified by rural-urban areas among older Mongolians based on a modified WHO-SAGE instrument**

| **Attributes** | | **Adjusted Odds Ratios [95% Confidence Interval]** | | | | |
| --- | --- | --- | --- | --- | --- | --- |
|  |  | **Rural areas** | | **Urban (*ger* and *apartment*) areas** | |  |
|  |  | **Outpatient use** | **Inpatient use** | **Outpatient use** | **Inpatient use** |  |
| **Sex** | Male | Reference | Reference | Reference | Reference |  |
|  | Female | **2.08** [1.29; 3.38] | 1.08 [0.67-1.73] | 1.60 [0.96; 2.66] | 1.54 [0.94; 2.53] |  |
| **Age group** | 60-69 | Reference | Reference | Reference | Reference |  |
|  | 70-79 | 0.92 [0.38; 2.28] | 0.49 [0.20; 1.20] | 1.07 [0.48; 2.37] | 1.22 [0.58; 2.69] |  |
|  | 80+ | 1.15 [0.22; 5.96] | 0.26 [0.05; 1.31] | 3.00 [0.62; 14.5] | 1.52 [0.34; 6.81] |  |
| **Marital status** | Married/Cohabiting | Reference | Reference | Reference | Reference |  |
|  | Not married/Divorced | 1.59 [0.58; 4.33] | 0.46 [0.17; 1.22] | 1.97 [0.58; 6.64] | 0.32 [0.09; 1.14] |  |
|  | Widowed | 1.17 [0.66; 2.08] | 0.76 [0.44; 1.34] | 0.86 [0.51; 1.43] | 0.89 [0.84; 1.46] |  |
| **Religion** | Buddhist | Reference | Reference | Reference | Reference |  |
|  | Other | 0.65 [0.37; 1.16] | 0.89 [0.51; 1.55] | 0.62 [0.41; 0.96] | 0.87 [0.57; 1.32] |  |
| **Ethnicity** | Mongol | Reference | Reference | Reference | Reference |  |
|  | Other | n/a | n/a | 0.56 [0.33; 1.01] | 1.32 [0.77; 2.26] |  |
| **Education** | Primary school or below | Reference | Reference | Reference | Reference |  |
|  | Secondary school | 1.18 [0.64; 2.16] | 1.17 [0.65; 2.11] | 0.61 [0.27; 1.35] | 1.36 [0.63; 2.97] |  |
|  | High school | 1.11 [0.58; 2.13] | 1.81 [0.95; 3.43] | 1.00 [0.60; 1.67] | 1.05 [0.64; 1.73] |  |
|  | Tertiary or higher | 1.67 [0.79; 3.57] | 1.63 [0.78; 3.38] | 0.59 [0.33; 1.06] | 1.57 [0.88; 2.80] |  |
| **Economic tertile** | Tertile 1 (lowest) | 0.73 [0.37; 1.46] | 1.52 [0.78; 2.96] | 0.60 [0.35; 1.03] | 1.05 [0.63; 1.78] |  |
|  | Tertile 2 | 1.09 [0.58; 2.08] | 0.85 [0.46; 1.59] | 1.56 [0.94; 2.59] | 0.52 [0.31; 0.87] |  |
|  | Tertile 3 (highest) | Reference | Reference | Reference | Reference |  |
| **Chronic conditions** | 0 | Reference | Reference | Reference | Reference |  |
|  | 1 | **2.32 [1.29; 4.20]** | **1.95 [1.10; 3.47]** | **2.23 [1.17; 4.58]** | **2.10 [1.01; 4.35]** |  |
|  | 2+ | **3.94 [2.22; 7.02]** | **3.06 [1.75; 5.35]** | **3.37 [1.63; 6.98]** | **3.38 [1.67; 3.80]** |  |

**Supplement Table2 Disparities in payment, travel mode, and travel time among older Mongolians based on a modified WHO-SAGE instrument**

|  | **Adjusted Odds Ratios for the same attributes as in Table 4 [95% Confidence Interval]** | | | |
| --- | --- | --- | --- | --- |
|  | **Payment (ref: free vs not free)** | **Travel mode (ref: private vehicle vs others)** | **Travel time (ref <1 hour vs ≥1hour)** |  |
| **Outpatient service use** |  |  |  |  |
| Rural | **3.65 [1.80; 7.43]** | **0.36** [0.16; 0.80] | 2.13 [0.89; 5.12] |  |
| *Urban-ger* | 0.98 [0.44; 2.17] | 0.79 [0.33; 1.89] | 0.69 [0.24; 2.05] |  |
| *Urban-apartment* | Reference | Reference | Reference |  |
| **Inpatient service use** |  |  |  |  |
| Rural | 1.46 [0.77; 2.76] | 0.52 [0.26; 1.07] | 1.80 [0.81; 4.03] |  |
| *Urban-ger* | 0.98 [0.51; 1.87] | 0.78 [0.37; 1.64] | 1.47 [0.65; 3.29] |  |
| *Urban-apartment* | Reference | Reference | Reference |  |
